# COVID-19 inhibits spermatogenesis in the testes by inducing cellular senescence

**DOI:** 10.3389/fgene.2022.981471

**Published:** 2023-01-05

**Authors:** Zuomin Wang, Yuxiang Ma, Zude Chen, Riwei Yang, Qinwei Liu, Jinyou Pan, Jiamin Wang, Yangzhou Liu, Mingda Zhou, Yihan Zhang, Yuhao Zhou, Shuxin Yang, Bangyu Zou, Jingwei Lin, Yingxin Cai, Zheng Jiang, Zhen Zhou, Zhigang Zhao

**Affiliations:** Department of Urology and Andrology, Minimally Invasive Surgery Center, Guangdong Provincial Key Laboratory of Urology, The First Affiliated Hospital of Guangzhou Medical University, Guangzhou, Guangdong, China

**Keywords:** COVID-19 (SARS-CoV-2), cellular senescence, spermatogenesis, cholesterol and testosterone biosynthesis, lactate and pyruvate synthesis

## Abstract

**Introduction:** COVID-19 (SARS-CoV-2) has been linked to organ damage in humans since its worldwide outbreak. It can also induce severe sperm damage, according to research conducted at numerous clinical institutions. However, the exact mechanism of damage is still unknown.

**Methods:** In this study, testicular bulk-RNA-seq Data were downloaded from three COVID-19 patients and three uninfected controls from GEO to evaluate the effect of COVID-19 infection on spermatogenesis. Relative expression of each pathway and the correlation between genes or pathways were analyzed by bioinformatic methods.

**Results:** By detecting the relative expression of each pathway and the correlation between genes or pathways, we found that COVID-19 could induce testicular cell senescence through MAPK signaling pathway. Cellular senescence was synergistic with MAPK pathway, which further affected the normal synthesis of cholesterol and androgen, inhibited the normal synthesis of lactate and pyruvate, and ultimately affected spermatogenesis. The medications targeting MAPK signaling pathway, especially MAPK1 and MAPK14, are expected to be effective therapeutic medications for reducing COVID-19 damage to spermatogenesis.

**Conclusion:** These results give us a new understanding of how COVID-19 inhibits spermatogenesis and provide a possible solution to alleviate this damage.

## Introduction

COVID-19 has been spreading throughout the world since December 2019. Globally, up to May 2022, there have been more than 520,000,000 confirmed cases of COVID-19, including 6,000,000 deaths, reported to WHO (https://covid19.who.int/).

COVID-19 invades the human body through ACE2 receptor and harms not only the respiratory system, but also various organs with high ACE2 expression, according to clinical data from multiple sites (Verdecchia et al., 2020; Walls et al., 2020). ACE2 is also abundant in the human testis, where it is mostly found in Sertoli cells and Leydig cells (Shen F et al., 2020). COVID-19 induces spermatogenesis damage, according to several studies, posing a major hazard to male reproductive health (Koç and Keseroğlu, 2021). COVID-19 is now infecting an increasing number of patients due to the introduction of many variants, posing a serious hazard to male reproductive health (Gul et al., 2021; Koç and Keseroğlu, 2021).

The normal spermatogenesis and sperm quality are the basis for human reproduction and transmission (de Kretser et al., 1998). Spermatogenesis is a complex process that results from the precise and orderly expression and interaction of different genes in different spatiotemporal programs under the action of different endocrine hormones and paracrine factors in spermatogenic cells, and is regulated by neuroendocrine and local regulation (de Kretser et al., 1998; Neto et al., 2016).

Androgen is a steroid hormone mainly produced by the endoplasmic reticulum of testicular Leydig cells. The main ingredient of androgen is testosterone, the most important hormone that initiates and maintains spermatogenesis (Smith and Walker, 2014). The conversion from cholesterol to progestenolone is the first step in the formation of all steroid hormones. Androgen and estrogen are all converted from cholesterol generated by cytochrome P450scc. Therefore, normal cholesterol metabolism plays an important role in normal testosterone synthesis in the testis (Miller, 2002).

During spermatogenesis, spermatogenic cells at all stages are found in the gap between Sertoli cells, and spermatogonia, spermatocyte, spermatocyte and sperm form the basement membrane to the cavity (de Kretser et al., 1998). Various nutrients are transported to spermatogenic cells by Sertoli cells (Rato et al., 2012). Under the influence of FSH, Sertoli cells make and give energy in the form of lactate and pyruvate during the maturation of spermatogenic cells. The overall lactate synthesis of Sertoli cells affects the ATP production required for meiosis in primary spermatocytes and sperm cells (Boussouar and Benahmed, 2004; Oliveira et al., 2015). Glycolysis is the body’s principal pyruvate and lactic acid synthesis pathways, implying that adequate glycolysis is necessary for normal spermatogenesis (Jutte et al., 1981).

There is mounting evidence that COVID-19 has a negative impact on spermatogenesis (Koç and Keseroğlu, 2021). Because male reproductive health is increasingly jeopardized as a result of increasingly serious COVID-19 situations, especially in more nations where COVID-19 is “co-existing,” we should pay more attention to the negative impacts of COVID-19 on the male reproductive system. We now have a limited grasp of how COVID-19 influences the spermatogenesis. As a result, we’ll use bioinformatics to investigate the impact of COVID-19 on spermatogenesis and look for suitable medications to mitigate the damage.

## Materials and methods

### Finding differential genes (DEGs)

In order to explore how COVID-19 affects the spermatogenesis, We looked for all the datasets about the impact of COVID-19 on spermatogenesis in the GEO database. The testicular bulk-RNA-seq data from three COVID-19 patients and three uninfected controls were downloaded in the NCBI GEO database (PRJNA661970). Identification of DEGs Analysis of gene expression between samples from three COVID-19 patients and three uninfected controls from GEO was conducted using “limma” package (Diboun et al., 2006) in R software. We defined DEGs as fold-change ≥ 2 and *p*-value < .001 was considered statistically significant.

### GO, KEGG and WikiPathways pathway enrichment analysis

GO [Ashburner et al., 2000; The Gene Ontology (GO) project in 2006, 2006] is a common and useful method that can enrich the biological functions of differential genes, including biological processes (BPs), cell components (CCs), and molecular functions (MFs). Kyoto Encyclopedia of Genes and Genomes (KEGG) (Kanehisa and Goto, 2000) is a database that integrates genome, chemistry and system function information. WikiPathways (WP) (https://www.wikipathways.org)) (Martens et al., 2020) is a biological pathway database known for its collaborative nature and open scientific methods. In order to obtain more accurate functional enrichment information of Go, KEGG and WikiPathways, clusterprofiler (Yu et al., 2012) software package of R platform was used to perform functional enrichment analysis of Go, KEGG and WikiPathways. The screening standard *p*-value < .05 was adjusted.

The calculation of *p*-value of differential gene enrichment [The Gene Ontology (GO) project in 2006, 2006] is based on hypergeometric method. The formula is:

$$P=1-\overset{m-1}{\underset{i=0}{\sum }}\frac{\left(\begin{array}{c} M\\ i \end{array}\right)\left(\begin{array}{c} N-M\\ n-i \end{array}\right)}{\begin{array}{c} N\\ n \end{array}}$$

*N* is the number of genes with Pathway annotation in all genes; *n* is the number of differentially expressed genes in *N*; *m* is the number of genes annotated as a particular Pathway in all genes; m represents the number of differentially expressed genes annotated as a specific Pathway. The calculated *p*-value will be further corrected by multiple tests to obtain corrected *p*-value. Usually, we use adj *p*-value < .05 as the threshold, and the pathway satisfying this condition is defined as the pathway that is significantly enriched in differentially expressed genes.

### ssGSEA is used to calculate the enrichment of the entire pathway or gene set

Single sample gene set enrichment analysis (ssGSEA) (Barbie et al., 2009)ranks the gene expression values of a given sample according to the absolute expression (in a sample). Then determine whether the members of a particular path are at the top or bottom of the list, indicating that they are related to phenotypic differences rather than evenly or randomly distributed in the list. Next, in order to evaluate this “enrichment” level, Enrichment Scores (ES) are calculated to quantify the over-expression of the set s above or below the entire ranking list L. Specific calculation method: The enrichment fraction was obtained by integrating the differences between ECDFs (Emperical Cumulative Density Function). For a given signature G of size NG and single sample S, of the dataset of N genes, the genes are replaced by their ranks according their absolute expression L = {r1, r2… rN}. The list is then ordered from the highest rank N to the lowest 1. An enrichment score ES (G, S) is obtained by a sum (integration) of the difference between a weighted ECDF of the genes in the signature PwG and the ECDF of the remaining genes PNG:

$$ES\left(G,\;S\right)=\sum_{i=1}^{N}\left[P_{G}^{w}\left(G,\;S,\;i\right)-P_{NG}\left(G,\;S,\;i\right)\right]$$

$$whereP_{G}^{w}\left(G,\;S,\;i\right)=\sum_{rj\in G,j\leq i}\frac{{\left|rj\right|}^{\alpha }}{\underset{rj\in G}{\sum }{\left|rj\right|}^{\alpha }}andP_{NG}\left(G,\;S,\;i\right)=\sum_{rj\notin G,j\leq i}\frac{1}{\left(N-N_{G}\right)}$$

The “GSVA” (Subramanian et al., 2005) software package of the R platform was used to ssGSEA analysis, and the screening criterion was adjusted *p*-value < .05.

### Protein–protein interaction networks was used to identify hub genes

Protein–protein interaction networks is a database of known and predicted protein–protein interactions (STRING; 10.5; http://www.string-db.org/) (Franceschini et al., 2013). It currently contains approximately 24.6 million proteins from 5,090 organisms. In the present work, potential target interactions were analyzed by using String with the settings of Organism for “*Homo sapiens*” and a confidence score ≥ .9. The PPI network of DEGs was visualized by Cytoscape (version 3.5.0) and the plug-in Molecular Complex Detection (MCODE) was used to screen functional modules to identify hub genes. A *p*-value < .05 was considered statistically significant.

Build a cross-network to study the interaction sites between pathways using topGO (Alexa et al., 2006). Correlation analysis were performed with the rcorr package in Hmisc in R (version 4.03. http://www.R-project.org/).

## Result

1) Many biological processes were harmed as a result of COVID-19 infection, including spermatogenesis

Various organs in the body were severely damaged after being infected by COVID-19 through ACE2 receptor. The testis, which has a high level of ACE2 expression in the body, cannot be spared (Verdecchia et al., 2020). Previous researches had suggested that most males with COVID-19 infection have lower sperm quality (Koç and Keseroğlu, 2021).

Many biological processes *in vivo* will be considerably impacted after COVID-19 infection. By downloading GEO’s bulk-RNA-seq data from three COVID-19 patients and three uninfected controls, Differential genes between the two groups were screened, and differential genes function enrichment analysis was performed to better understand how COVID-19 impacts spermatogenesis in the human testis.

We used the R package “LIMMA” to examine the differences genes between the two groups. Analysis revealed 15850 mRNAs in the testes of controls and COVID-19 patients, of which 2,645 were up-regulated while 2,789 were down-regulated (folding change > 2, FDR < .001). These findings imply that during specific biological processes, COVID-19 infection triggers dynamic alterations at the molecular level. In order to further explore the changes of testicular biological processes after COVID-19 infection, we carried out KEGG, GO, WIKIPATHWAYS functional enrichment analysis of differential genes.

**FIGURE 1:**
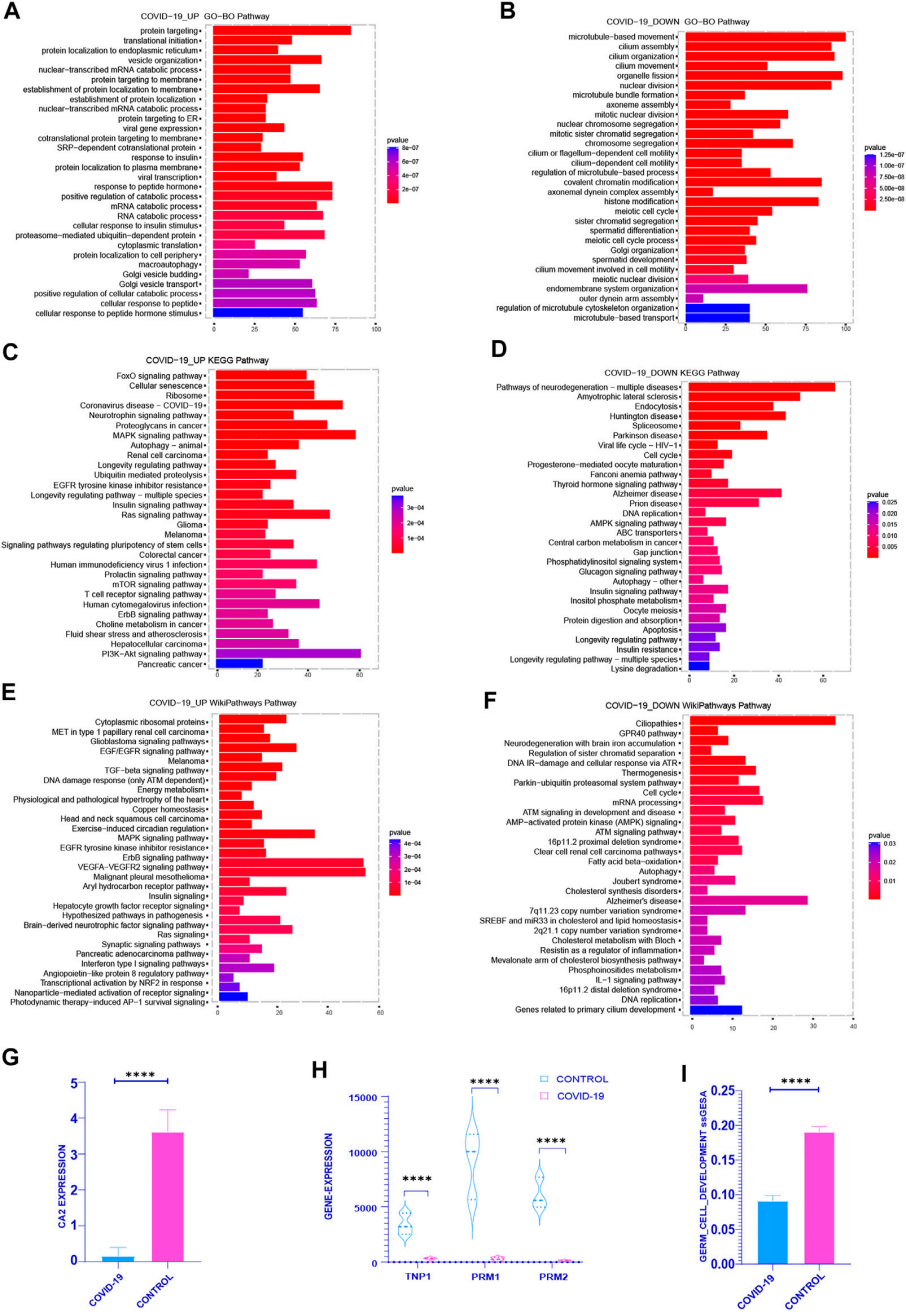
Functional enrichment analysis was performed on the differential genes between the COVID-19 group and the control group (including the increased gene set and the decreased gene set). **(A,C,E)** gene sets were enriched in GO, KEGG, WIKIPATHWAYS, respectively, and the top 30 enrichment pathways were selected according to Adj-p-Value from small to large. **(B,D,F)** decreased gene sets were enriched in GO, KEGG, WIKIPATHWAYS, respectively, and the top 30 enrichment pathways were selected according to Adj-p-Value from small to large. **(G)** The expression of several typical sperm Markers: TNP1, PRM1 and PRM2 decreased significantly in COVID-19 group (*p* <.001). **(H)** The expression of classical Marker: CA2 decreased after COVID-19 infection (*p* < .001). **(I)** The ssGSEA score of Germ Cell Development pathway decreased in COVID-19 group (*p* < .001).

First of all, in order to definite the functional enrichment of differential genes, we choose the pathway with the top 30 adj *p*-value of the enrichment pathway to better highlight the effect of COVID-19 on the testis. In the upregulated genes after COVID-19 infection, GO enrichment mainly concentrated in: Viral transcription, Viral gene expression, Response to insulin, Response to peptide hormone, Translation initiation (Figure 1A). FoxO signaling pathway, Cellular senescence, Coronavirus disease-COVID-19, MAPK signaling pathway, Longevity regulation pathway-multiple species, and PI3K-Akt signaling pathway are all heavily represented in KEGG (Figure 1C). In the WIKIPATHWAYS pathway, it is mainly enriched in EGF/EGFR signaling, TGF-beta signaling, DNA damage response (only ATM dependent), MAPK signaling, EGFR tyrosine kinase inhibitor resistance, Ras signaling (Figure 1E). The genes that downregulated following infection with COVID-19 were mostly focused in: GO functional enrichment: Sister chromatid segregation in mitosis, Chromosomal segregation, Cell motility mediated by the cilium or flagellum, Meiotic cell cycle, Spermatid differentiation, Meiotic cell cycle process (Figure 1B). Cell cycle, DNA replication, AMPK signaling pathway, Insulin signaling pathway, Oocyte depletion, Longevity regulation pathway, and so on are all included in the KEGG database (Figure 1D). WIKIPATHWAYS database: Regulation of sister chromatid separation at the metaphase-anaphase transition, Thermogenesis, Cell cycle, AMP-activated protein kinase (AMPK) signaling, Fatty and Kandutsch-Russell pathways, DNA replication, and other topics (Figure 1F). Upregulated genes were primarily enriched in Coronavirus disease-COVID-19, viral replication, cell senescence, MAPK and other signaling pathways in COVID-19-infected patients, while down-regulated genes were primarily enriched in chromosome separation, reduced division and sperm differentiation. At the same time, after COVID-19 infection, cell cycle was enriched in down-regulated genes. The enrichment of these pathways showed that COVID-19 may affect many normal biological processes *in vivo*.

Therefore, we explored the COVID-19 patient’s sperm differentiation level. The important enzyme CA2 in the sperm differentiation process was considerably reduced in COVID-19 group (Figure 1G). At the same time, the expression of the classical marker genes TNP1, PRM1 and PRM2 of sperm were reduced in COVID-19 (Figure 1H). The ssGSEA score of Germ Cell Development pathway also decreased significantly after COVID-19 infection (Figure 1I). From these, we can draw a conclusion: after COVID-19 infection, it affects the spermatogenesis through a variety of biological processes, and ultimately affects the quantity and quality of sperm, which poses a great threat to the reproductive health of male.

2) COVID-19 inducing senescence in testicular cells after COVID-19 infection

Based on the previous analysis of the top 30 pathways in GO, KEGG, WIKIPATHWAYS functional enrichment pathway of the differentially expressed genes up and down after COVID-19 infection, we can see that cell cycle related pathways [cell cycle, DNA replication, Neurodegeneration with brain iron accumulation (NBIA) subtypes pathway, etc.] occur repeatedly in GO, KEGG, WP enrichment pathways of downregulated gene sets (Figures 1B, D, F). Multiple signaling pathways affecting cell cycle are concentrated in the up-regulated gene sets (Cellular senescence, MAPK signaling pathway, Longevity regulation pathway-multiple species, Ras signaling pathway, mTOR signaling pathway, PI3K-Akt signaling pathway) (Figures 1A, C, E). We consider whether COVID-19 affects spermatogenesis by influencing cell cycle-related pathways.

**FIGURE 2:**
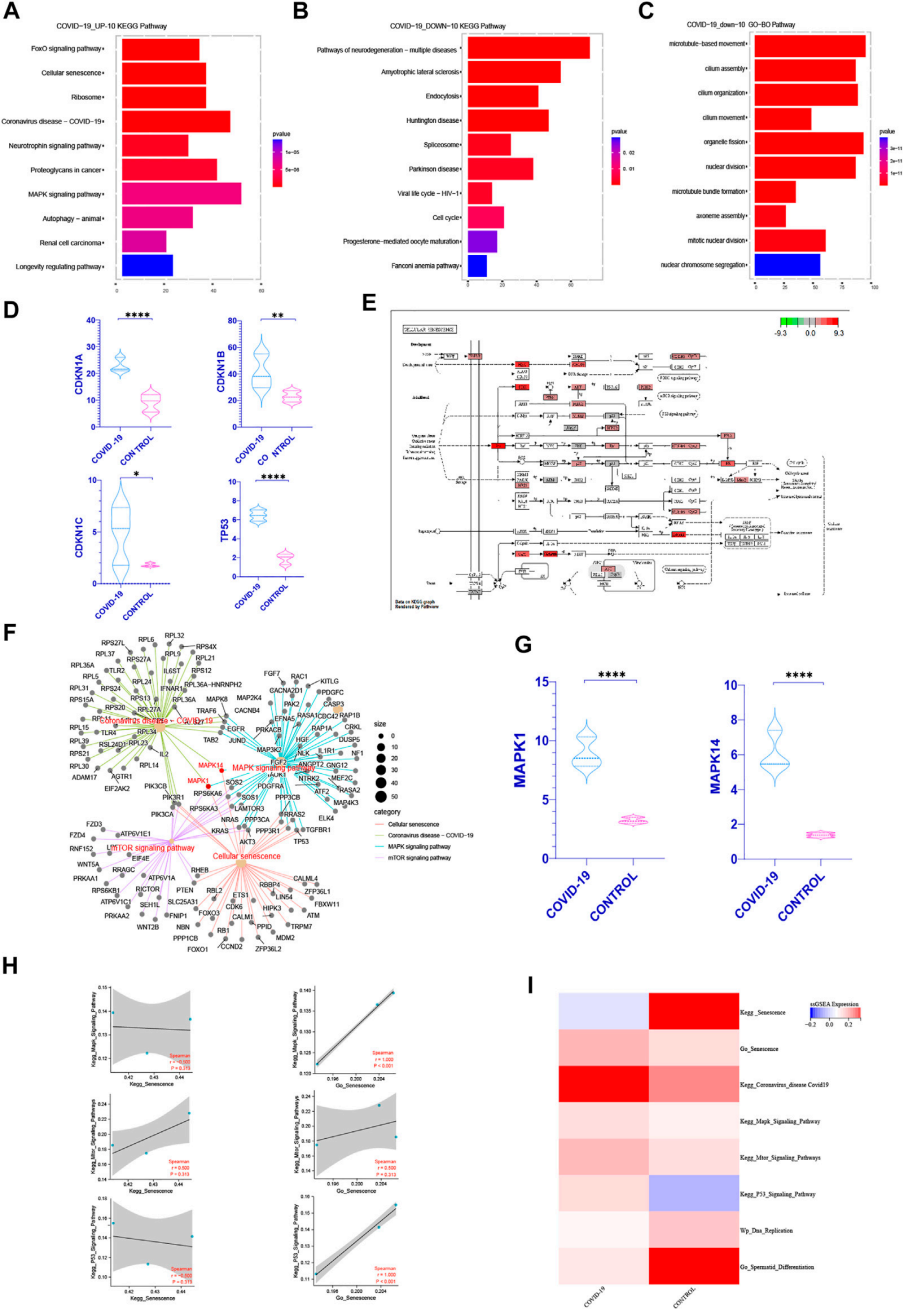
COVID-19 inducing senescence in testicular cells after COVID-19 infection. **(A)** After KEGG enrichment of the significantly increased genes in patients with COVID-19 infection, the first 10 pathways with the most obvious *p*-value. Cellular senescence is the most obvious position of *p*-value (FOXO pathway is one of the internal pathways of cell senescence pathway) **(B,C)** After KEGG, GO enrichment of genes significantly decreased in patients with COVID-19 infection, the first 10 pathways with the most significant *p*-value were obtained. Cell cycle recurred among them **(D)** Cellular senescence classic Markers: CDKN1A, CDKN1B, CDKN1C, TP53 increased in COVID-19 group (*p* < .05). **(E)** The enrichment of enrichment sites in the internal pathway of KEGG—Senescence was detailed, and mTOR, Mapk, and P53signaling pathway were enriched significantly. **(F)** Establishing a cross-network between mTOR, Mapk, P53 signaling pathway and Coronavirus disease-COVID-19, we can see that MAPK1, MAPK14 play important role as a bridge. **(G)** The gene expression of MAPK1 and MAPK14 were significantly increased in COVID-19 group. **(H)** Correlation between Kegg Senescence Pathway and GO Senescence Pathway and other related pathways that inhibit cell cycle. GO Senescence Pathway is more positively correlated with them. **(I)** ssGSEA score of Kegg Senescence Pathway, GO Senescence Pathway, sperm differentiation, DNA replication and other cell cycle inhibition pathways. ssGSEA score of GO Senescence Pathway and other cell cycle inhibition pathways increased.

In order to better understand how COVID-19 negatively affects patients’ spermatogenesis, we concentrate on the most significant pathways especially cell cycle related ones. Interestingly, in the top 10 pathways of KEGG enrichment within the up-regulated genes after infected with COVID-19, cellular senescence pathway is the most significant one (Figure 2A). At the same time, we discovered that following COVID-19 infection, the expression of key genes CDKN1A, CDKN1B, CDKN1C, and TP53 in cellular senescence rose dramatically (Figure 2D). The cell cycle occupied a prominent position in the top 10 pathways of KEGG functional enrichment in the genes that downregulated following COVID-19 infection (Figure 2B). In the top 10 pathways of GO and WIKIPATHWAYS functional enrichment of the down-regulated genes, the cell cycle and nuclear chromosomal separation pathways have been enriched (Figure 2C, Supplementary Figure S2). This suggests that COVID-19 may affect the cell cycle by inducing cellular senescence.

To confirm our hypothesis, we first found out pathways linked to cellular senescence based on past researches, such as the mTOR, MAPK, P53 signaling pathway (Rufini et al., 2013; Houssaini et al., 2018; Papaconstantinou, 2019). At the same time, we can detect the enrichment of these three pathways in the KEGG senescence pathway internal enrichment (Figure 2E). We looked at the relative levels of ssGSEA expression in different cell senescence related pathways. It shows that during COVID-19 infection, the mTOR, MAPK, P53 signaling pathway increased, but DNA replication and sperm differentiation reduced dramatically (Figure 2I). These pathways all indicated that COVID-19 infection may induce testicular cell senescence, affect the normal cell cycle. We also built a cross-network that included the cellular senescence pathway, the mTOR pathway, the MAPK signaling pathway and the Coronavirus disease-COVID-19. It can be seen that MAPK1, MAPK14 play important bridging role (Figure 2F) and increase significantly in patients with COVID-19 infection (Figure 2G). These also provides a molecular basis for COVID-19 inducing cellular senescence. In conclusion, cellular senescence may be one of the most significant changes in the testis of patients with COVID-19 infection. Exploring the role of cellular senescence may help us better understand the effects of COVID0-19 on testicular physiological processes, especially on spermatogenesis.

In order to further explore how COVID-19 induces cellular senescence and how cellular senescence further affects other pathways, we scored the relevant biological signal pathways by ssGSEA. The method for calculating the ssGSEA score and the enrichment significance of differential genes are explained in detail in method. Through the analysis of the ssGSEA scoring method and the enrichment scores of GO, KEGG, WIKIPATHWAYS, we can see that the higher the specificity of genes in a specific pathway, the less the number of genes, the more beneficial to the enrichment significance of differential genes such as GO, KEGG, WIKIPATHWAYS, On the other hand, ssGSEA means that the more accurate the number of related genes in a specific pathway, the more extreme the gene distribution is, and the more obvious the score is.

It is well known that there are many senescence-related genes. We can see from the analysis of KEGG Senescence Pathway and GO Senescence Pathway that KEGG Senescence Pathway mainly includes 19 senescence-related genes, while GO Senescence Pathway contains 130 senescence-related genes. This may occur when the senescence-related genes in KEGG Senescence Pathway are used for ssGSEA scoring, and the scores related to senescence genes obtained will be offset by the scores of genes that are not effectively included in the KEGG Senescence Pathway but are closely related to senescence, and ultimately affect the scoring results. This inference can be verified in the ssGSEA scoring of KEGG Senescence Pathway and GO Senescence Pathway (Figure 2I). At the same time, the correlation between the ssGSEA scores of the two and the ssGSEA scores of the pathways related to cell cycle inhibition was analyzed. It can be seen that the ssGSEA scores of GO Senescence Pathway were significantly positively correlated with the ssGSEA scores of the pathways related to cell cycle inhibition, which was consistent with previous studies, while the ssGSEA scores of KEGG Senescence Pathway were weakly positively correlated or even negatively correlated (Figure 2H). Therefore, in the subsequent ssGSEA scoring and correlation analysis between pathways, we choose GO senescence pathway to explore.

3) COVID-19 induced testicular cell senescence through MAPK signaling pathway

It is well known that cellular senescence is a special cell state and is characterized by slow proliferation and cell cycle arrest (Muñoz-Espín and Serrano, 2014). Cellular senescence is caused by a variety of factors, including 1) DNA damage-induced senescence 2) Oncogene-induced senescence 3) Oxidative stress-induced senescence 4) Chemotherapy-induced senescence 5) Mitochondrial dysfunction-associated senescence (MiDAS) 6) Epigenetically induced senescence 7) Paracrine senescence (Hernandez-Segura et al., 2018).

**FIGURE 3:**
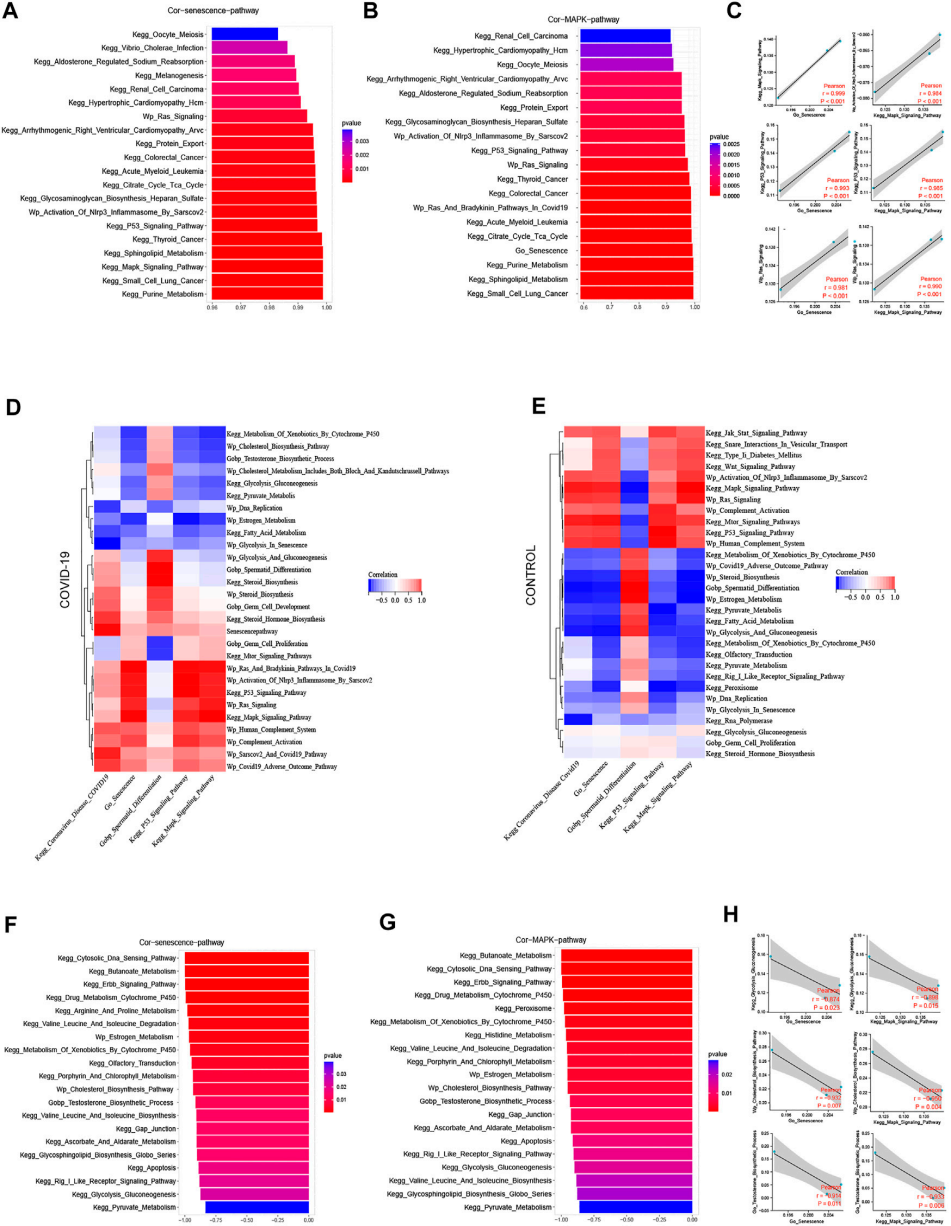
Screening pathways associated with cellular senescence during COVID-19 infection. **(A)** The top 20 pathways in COVID-19 were significantly positively correlated with cellular senescence, among which MAPK signaling pathway was the most relevant (*r* = .999, *p* < .001). **(B)** The top 20 pathways that were significantly positively correlated with MAPK signaling pathways are similar to those related to cellular senescence. **(C)** Cellular senescence and MAPK signaling pathways are roughly consistent with cell cycle-related pathways. **(D)** The correlation of cellular senescence with GO gene sets, KEGG pathways, and WIKIPATHWAYS pathways in COVID-19 group. **(E)** The correlation of cellular senescence with GO gene sets. KEGG pathways, and WIKIPATHWAYS pathways in the Control group. **(F)** The top 20 pathways in COVID-19 were negatively correlated with cellular senescence (*r* < −.8, *p* < .001). These pathways mainly function in cholesterol and testosterone synthesis, as well as glycolysis and pyruvate metabolism. **(G)** COVID-19 were significantly negatively correlated with MAPK signaling pathway (*r* < −.8, *p* < .001), and their main enrichment pathways were similar to those related to cellular senescence **(H)** Cellular senescence and MAPK signaling pathway were roughly consistent with testosterone synthesis, cholesterol synthesis and glycolysis pathway.

We examined the top 20 signaling pathways significantly positively linked with the cellular senescence in COVID-19 patients to further understand how COVID-19 induces testicular cell senescence. These pathways can be found mostly in the KEGG MAPK Signaling Pathway, KEGG P53 Signaling Pathway, Wp Activation of Nlrp3 Inflammasome by Sars-cov2 and Wp Ras Signaling (Figure 3A). Among them, MAPK signal pathway was the most correlated one, which was positively correlated with cellular senescence pathway (*r* = .999, *p* < .001).

MAPK signaling pathway is mainly composed of four subfamilies, namely, 1) Extracellular-signal-regulated protein kinase (ERK), 2) P38 mitogen-activated protein kinase (p38 MAPK), 3) C-Jun N-terminal kinase (JNK), 4) Extracellular signal-regulated kinase 5 (ERK5). These MAPK subfamilies are participate in a variety of signal transduction pathways, including ERK, which regulates cell growth and differentiation, and p38 MAPK signaling pathways (Kim and Choi, 2010), which are involved in stress responses like inflammation and apoptosis. The above two subfamily pathways as part of the inducement of inducing cellular senescence (Figure 1E).

We looked at the relationship between the MAPK signaling pathway and other pathways in COVID-19 patients in this study. In the COVID-19 group, MAPK pathway was significantly positively correlated with Coronavirus disease COVID-19, and negatively correlated with sperm differentiation. Cell senescence and Coronavirus disease COVID-19 and sperm differentiation also have the same trend with MAPK pathway (Figure 3D). We investigated the signaling pathway closely correlated to the MAPK pathway in patients with COVID-19 infection to further understand its probable mechanism. The KEGG P53 Signaling Pathway, Wp Activation of Nlrp3 Inflammasome by Sarscov2, Wp Ras Signaling, Go Senescence, and MAPK pathways are all significantly positively correlated in patients with COVID-19 infection, and these pathways are highly consistent with the pathways that are positively correlated with cellular senescence (Figure 3B). At the same time, we discovered a strong link between cell cycle inhibition related signaling pathways and cellular senescence signaling pathway, as well as MAPK signaling pathway (Figure 3C). Combining the two subfamily MAPK pathways as part of the inducement of inducing cellular senescence (Figure 1E), it is suggested that MAPK pathway may promote testicular cellular senescence during COVID-19 infection.

A range of biological processes in the testis were affected when COVID-19 infection induced testicular cell senescence *via* the MAPK signaling pathway. The top 20 pathways that most negatively correlated with cellular senescence were KEGG Cytosolic DNA Sensing Pathway, KEGG Cytosolic DNA Sensing Pathway, KEGG Cytosolic DNA Sensing Pathway, KEGG Butanoate Metabolism, KEGG Erbb Signaling Pathway, KEGG Drug Metabolism Cytochrome P450, Wp Estrogen Metabolism, Wp Cholesterol Biosynthesis Pathway, Wp Cholesterol Biosynthesis Process, KEGG Gap Junction, KEGG Gly Pyruvate Metabolism KEGG etc. (Figure 3F). What were closely related to spermatogenesis in these pathways were cholesterol and testosterone synthesis, as well as glycolysis and pyruvate metabolism. Simultaneously, we discovered that the pathways, negatively correlated with both MAPK pathway and cellular senescence, are strikingly similar, particularly regarding: KEGG Drug Metabolism Cytochrome P450, KEGG Cytosolic DNA Sensing Pathway, KEGG Erbb Signaling Pathway, KEGG Butanoate Metabolism Wp Estrogen Metabolism, Wp Cholesterol Biosynthesis Pathway, KEGG Histidine Metabolism, KEGG Porphyrin And Chlorophyll Metabolism, KEGG Histidine Metabolism, KEGG Porphyrin And Chlorophyll Metabolism KEGG Gap Junction, KEGG Apoptosis, KEGG Glycolysis Gluconeogenesis, KEGG Pyruvate Metabolism, and others (Figure 3G).

MAPK pathway and cellular senescence exhibit a Similar negative correlation with testosterone synthesis, cholesterol synthesis, glycolysis and pyruvate metabolism (Figure 3H). Through the signaling pathway significantly negatively correlated with cellular senescence and MAPK signaling pathway, we speculated that after COVID-19 infection induced testicular cell senescence through MAPK pathway, whether cellular senescence could further affect androgen synthesis, normal glycolysis and pyruvic acid synthesis in testis, thereby affecting the spermatogenesis of patients has become a question worth exploring. This may shed fresh light on how COVID-19 suppresses spermatogenesis.

4) The Prominent Negative Role of Cellular senescence on Androgen Synthesis

**FIGURE 4:**
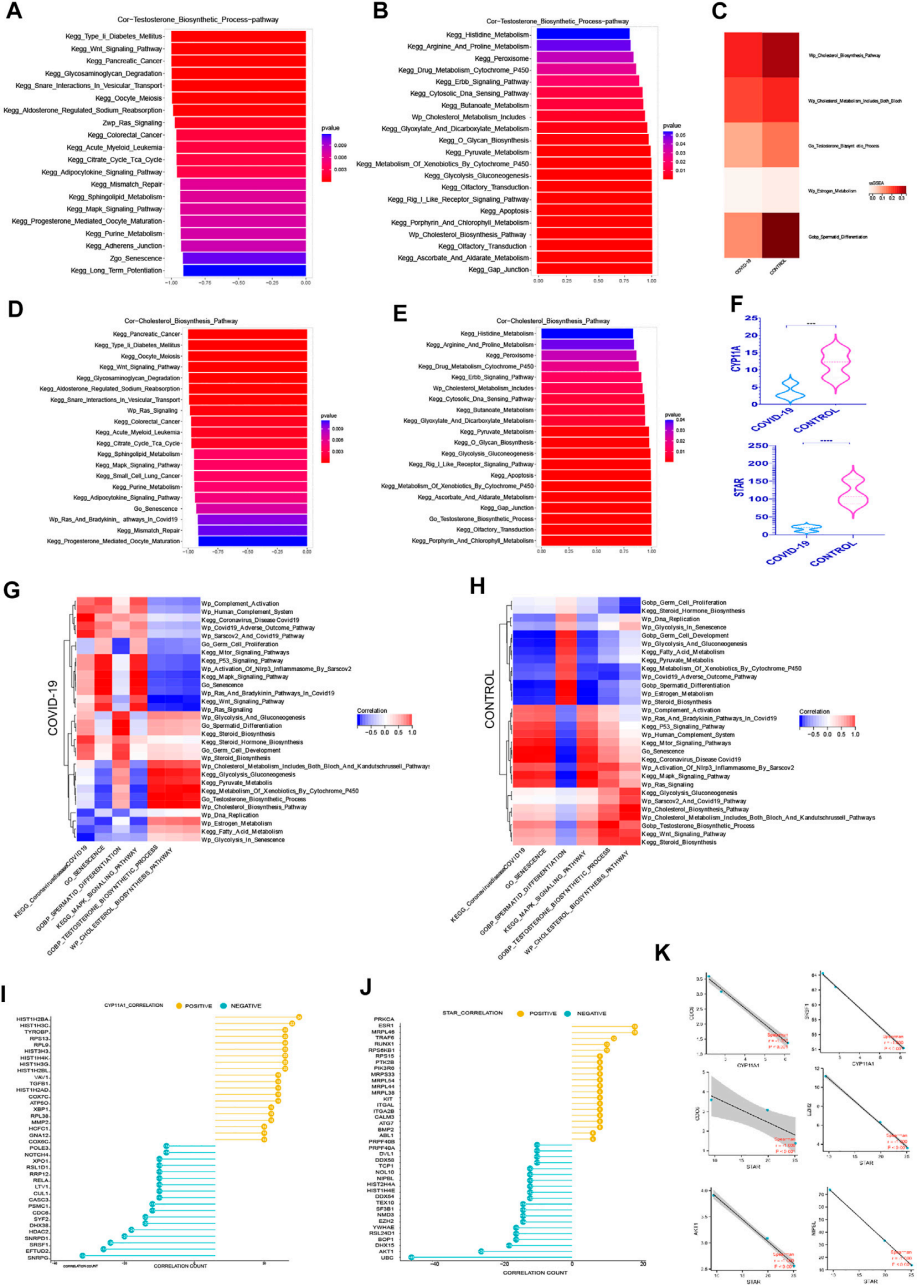
The Prominent Negative Role of Cellular senescence on Androgen Synthesis **(A)** The top 20 pathways in COVID-19 were significantly negatively correlated with testosterone synthesis pathway, among which Go Senescence pathway was the most relevant (*r* = −.91, *p* < .001). **(B)**The top 20 pathways in COVID-19 were significantly positively correlated with testosterone synthesis pathway, among which Cholesterol Biosynthesis Pathway play an importance role in those.**(C)** ssGSEA score of Wp Cholesterol Biosynthesis Pathway, Wp Cholesterol Metabolism,testosterone synthesis pathway decreased in COVID-19.**(D)** The top 20 pathways in COVID-19 were significantly negatively correlated with cholesterol synthesis pathway, among which Go Senescence pathway was the most relevant (*r* = −.93, *p* < .001). **(E)** The top 20 pathways in COVID-19 were significantly positively correlated with cholesterol synthesis pathway. **(F)** These two rate-limiting enzymes for cholesterol synthesis decreased in COVID-19 infection (STAR, CYP11A1). **(G)** The correlation of testosterone synthesis pathway, cholesterol biosynthesis Pathway with GO gene sets, KEGG pathways, and WIKIPATHWAYS pathways in COVID-19 group. **(H)** The correlation of testosterone synthesis pathway, cholesterol biosynthesis Pathway with GO gene sets, KEGG pathways, and WIKIPATHWAYS pathways in the Control group.**(I)** The top 20 genes strong negative correlation with STAR (*r* < −.9, *p* < .05) by PPI and the top 20 genes strong positive correlation with STAR (*r* > .9, *p* < .05) by PPI. **(J)** The top 20 genes strong negative correlation with CYP11A1 (*r* < −.9, *p* < .05) by PPI and the top 20 genes strong positive correlation with CYP11A1 (*r* > .9, *p* < .05) by PPI. **(K)** Several genes implicated in cell senescence are among the top 20 genes negatively correlated with STAR or CYP11A1.

Androgen synthesis is well recognized to be converted from cholesterol, and adequate androgen synthesis is critical for spermatogenesis (Miller, 2002; Smith and Walker, 2014). The question of whether testicular cell senescence influences androgen production has sparked debate. Among the androgens, testosterone, as the main active component, plays a key role in their physiological function (Smith and Walker, 2014). In order to reduce the interference of other androgen components, we focused on the testosterone hormone synthesis pathway. We can observe from the ssGSEA score of associated pathways that both testosterone and cholesterol synthesis decreased following COVID-19 infection, which was consistent with the decrease in Spermatid Differentiation (Figure 4C).

We evaluated the signaling pathways closely associated to cholesterol and testosterone synthesis, specifically the signaling pathways strongly related to cell cycle, to better understand how they regulate the synthesis of cholesterol and testosterone. In the top 20 most negatively correlated pathways related to testosterone synthesis pathway, we can see that KEGG Wnt Signaling Pathway, Wp Ras Signaling, KEGG Citrate Cycle Tca Cycle, KEGG MAPK Signaling Pathway, Go Senescence, KEGG Long Term Potentiation are significantly negatively correlated, which are mainly concentrated in the pathways related to cell cycle inhibition (Figure 4A). Between Go Senescence and testosterone synthesis pathway, the correlation coefficient was −.91, indicating a strong negative association. We can see that KEGG Drug Metabolism Cytochrome P450, Wp Cholesterol Metabolism Includes Both Bloch and Kandutschrussell Pathways, KEGG Pyruvate Metabolism, and KEGG Glycolysis Gluconeogenesis KEGG Apoptosis, Wp Cholesterol Biosynthesis Pathway were (Figure 4B) among the top 20 positive pathways correlated with the testosterone synthesis pathway. They were found functioning concentrated in the conversion of cholesterol to steroid hormones.

KEGG Wnt Signaling Pathway, KEGG Glycosaminoglycan Degradation, Wp Ras Signaling, KEGG Citrate Cycle Tca Cycle, KEGG MAPK Signaling Pathway, and Go Senescence were among the top 20 negative pathways correlated with the cholesterol synthesis pathway (Figure 4D), which Go Senescence and the cholesterol synthesis pathway had a correlation coefficient of −.93, indicating a strong negative correlation. KEGG Peroxisome, KEGG Drug Metabolism Cytochrome P450, Wp Cholesterol Metabolism Includes, KEGG Pyruvate Metabolism, KEGG Glycolysis Gluconeogenesis, KEGG Apoptosis, KEGG Xenobiotic Metabolism By Cytochrome P450, KEGG Gap Junction, and Go Testosterone Biosynthesis Process were among the top 20 positive pathways correlated with the cholesterol synthesis pathway (Figure 4E).

Consistent with expectations, the positive and negative correlation pathways of testosterone synthesis pathway and cholesterol synthesis pathway were highly similar, and the pathways that inhibited the normal cycle of cells had a significant negative correlation with the two, especially cellular senescence. Interestingly, pyruvate metabolism and glycolysis signaling pathways were positively correlated with both, suggesting that there was an indivisible relationship between effective energy metabolism and androgen synthesis.

Wp Cholesterol Metabolism Includes Both Bloch And Kandutschrussell Pathways, Wp Cholesterol Biosynthesis Pathway, KEGG Glycolysis gluconeogenesis Pathway were positively correlated with the testosterone synthesis pathway In both Control group and COVID-19 group (Figures 4G, H). It was claimed that adequate cholesterol synthesis and glycolysis were important in testicular cell testosterone synthesis. Multiple signaling pathways were altered as a result of COVID-19 infection. In the control group, KEGG mTOR Signaling Pathways, KEGG P53 Signaling Pathway, KEGG MAPK Signaling Pathway, Wp Human Complement System, Wp Complement Activation, Go Senescence, Wp Ras Signaling and KEGG Wnt Signaling Pathway were weakly negative or positive in relation to testosterone synthesis. However, after COVID-19 infection, the negative correlation with testosterone synthesis was significantly increased, indicating that after COVID-19 infection, normal testosterone synthesis was more and more closely related to normal cell cycle. The KEGG Pyruvate Metabolis, KEGG Metabolism Of Xenobiotics By Cytochrome P450 pathway were weakly negative with testosterone synthesis in the control group, but there was a significant positive correlation with testosterone synthesis after COVID-19 infection, demonstrating once again that normal pyruvate metabolism and cholesterol synthesis may played an important role in testosterone synthesis.

The difference in the expression level of STAR, CYP11A1 in androgen synthesis were analyzed to better understand the effect of COVID-19 on androgen synthesis. The two rate-limiting enzyme genes reduced dramatically after COVID-19 infection (Figure 4F), resulting in a decrease in overall androgen production after COVID-19 infection. Simultaneously, we looked for important genes that were positively and negatively correlated with the androgen synthesis rate-limiting enzyme, in order to learn more about the specific regulatory mechanism of androgen synthesis in COVID-19 infection. Protein–protein interaction networks (PPI) can more efficiently screen genes that play a major role in gene sets and clarify the associated mechanisms between genes. Firstly, 768 genes with strong negative correlation with STAR (*r* < −.9, *p* < .05) were detected by PPI on STRING website, and the top 20 genes were obtained. At the same time, PPI was applied to 800 positive correlated genes with STAR (*r* > .9, *p* < .05) to produce the top 20 genes (Figure 4I). PPI was also used to treat both the positive and negative linked genes of CYP11A1 (Figure 4J). Interestingly, several genes implicated in cell senescence are among the top 20 genes negatively correlated with STAR or CYP11A1 respectively, especially SRSF1, NIPBL expression changed dramatically during COVID-19, suggesting that SRSF1, NIPBL may be important in suppressing androgen synthesis during COVID-19-induced senescence Figure 4K.

5) The negative correlation between cellular senescence with pyruvate and lactate generation

Sertoli cells are crucial to the entire spermatogenesis. Under the influence of FSH, Sertoli cells produce a substantial amount of lactate and pyruvate, which they then pass to spermatogenic cells at all levels (Boussouar and Benahmed, 2004). Lactate and pyruvate produced by Sertoli cells are particularly important for the ATP required for meiosis in primary spermatocytes and sperm cells. Pyruvate and lactate can be transformed into each other *in vivo* by lactate dehydrogenase. The major mechanism for the synthesis of pyruvate and lactate *in vivo* is glycolysis, implying that adequate glycolysis is required for normal spermatogenesis (Jutte et al., 1981; Boussouar and Benahmed, 2004; Rato et al., 2012; Oliveira et al., 2015).

**FIGURE 5:**
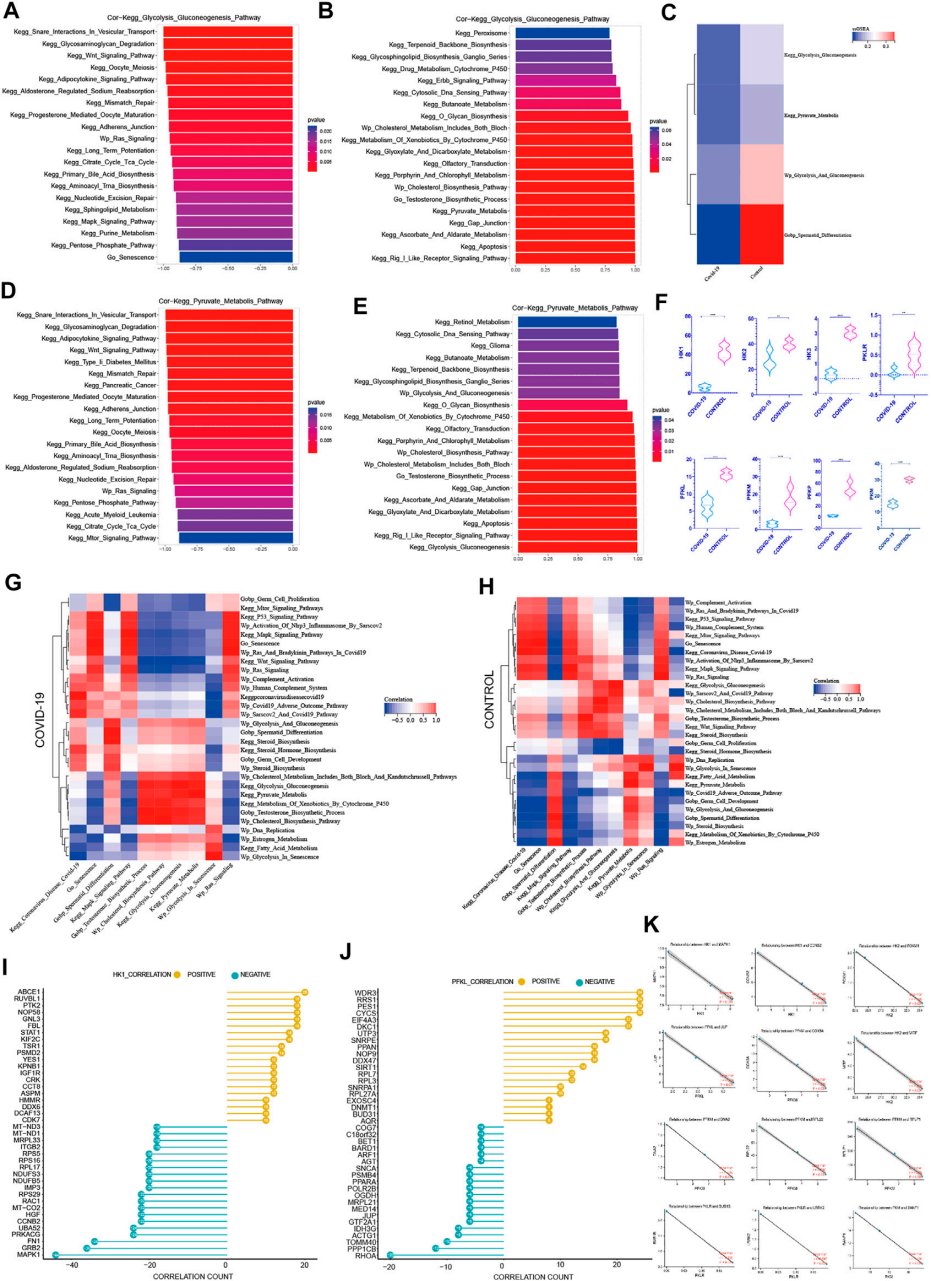
The negative correlation between cellular senescence with pyruvate and lactate generation **(A)** The top 20 pathways in COVID-19 were significantly negatively correlated with Kegg Glycolysis Gluconeogenesis, among which Go Senescence pathway was the most relevant (*r* = −.874, *p* < .001). **(B)** The top 20 pathways in COVID-19 were significantly positively correlated with Kegg Glycolysis Gluconeogenesis, which were mainly concentrated in the saccharometabolism-related pathways. **(C)** ssGSEA score of Kegg Glycolysis Gluconeogenesis, Kegg Pyruvate Metabolis, Wp Glycolysis And Gluconeogenesis decreased in COVID-19.**(D)** The top 20 pathways in COVID-19 were significantly negatively correlated with Kegg Pyruvate Metabolis, among which Go Senescence pathway was the most relevant (*r* = −.93, *p* < .001).**(E)** The top 20 pathways in COVID-19 were significantly positively correlated with Kegg Pyruvate Metabolis. **(F)** These three glycolytic rate-limiting enzymes decreased in COVID-19 infection (HK, PFKL, PKLR and their isoenzymes). **(G)** The correlation of Kegg Glycolysis Gluconeogenesis, Kegg Pyruvate Metabolis with GO gene sets, KEGG pathways, and WIKIPATHWAYS pathways in COVID-19 group. **(H)** The correlation of Kegg Glycolysis Gluconeogenesis, Kegg Pyruvate Metabolis with GO gene sets, KEGG pathways, and WIKIPATHWAYS pathways in the Control group.**(I)** The top 20 genes strong negative correlation with HK1 (*r* < −.9, *p* < .05) by PPI and the top 20 genes strong positive correlation with HK1 (*r* > .9, *p* < .05) by PPI. **(J)** The top 20 genes strong negative correlation with PFKL (*r* < −.9, *p* < .05) by PPI and the top 20 genes strong positive correlation with PFKL (*r* > .9, *p* < .05) by PPI. **(K)** Several genes implicated in cell senescence are among the top 20 genes negatively correlated with HK1, PFKL or PFKM.

Firstly, we focused on the rate-limiting enzymes in the glycolysis cycle and their expression differences. The three rate-limiting enzymes dropped after COVID-19 infection (Figure 5F). Then, using ssGSEA, we chose glycolysis-related signaling pathways from three datasets to compare the relative expression of glycolysis pathways in COVID-19 infection and control groups. The glycolysis signaling pathway and the pyruvate metabolism pathway both decreased following COVID-19 infection (Figure 5C), as expected. These meant that following COVID-19 infection, glycolysis metabolism was significantly inhibited. Inhibiting glycolysis reduced pyruvate synthesis and lactic acid synthesis, which subsequently affected the spermatogenesis unique energy metabolism, and lastly affected the spermatogenesis.

Among the top 20 most negative correlation pathways with glycolysis pathways, we can see that KEGG Wnt Signaling Pathway, Wp Ras Signaling, KEGG Long Term Potentiation, KEGG MAPK Signaling Pathway, Go Senescence, KEGG Purine Metabolism, KEGG Pentose Phosphate Pathway, and KEGG Oocyte Meiosis were negatively correlated. They were mainly concentrated in cell cycle inhibition related pathways (Figure 5A). The correlation coefficient between Go Senescence and glycolysis was −.874, showing a strong negative correlation. Among the top 20 positively correlated pathways in glycolysis pathways, we can see that KEGG Peroxisome, KEGG Glycosphingolipid Biosynthesis Ganglio Series, KEGG O Glycan Biosynthesis, KEGG Glyoxylate and Dicarboxylate Metabolism, KEGG Glyoxylate and Dicarboxylate Metabolism, KEGG Gap Junction, Wp Cholesterol Biosynthesis Pathway, Go Testosterone Biosynthesis Process were significantly positively correlated (Figure 5B), which were mainly concentrated in the saccharometabolism-related pathways.

Among the top 20 most negatively correlated pathways in the pyruvate metabolic pathway, KEGG Glycosaminoglycan Degradation, KEGG Type II Diabetes Mellitus, KEGG Adherens Junction, KEGG Oocyte Meiosis, Wp Ras Signaling Pathway, KEGG mTOR Signaling Pathway showed a significant negative correlation (Figure 5D). Among the top 20 most positively correlated pathways in the pyruvate metabolic pathway, KEGG Butanoate Metabolism, Wp Glycolysis And Gluconeogenesis, Wp Cholesterol Metabolism, Go Testosterone Biosynthesis Process, KEGG Gap Junction, KEGG Apoptosis were significantly positively correlated (Figure 5E). These showed that Cell cycle damage was significantly negatively correlated with normal glycolysis. Also Go Senescence was significantly negatively correlated with glycolysis pathway. Pyruvate metabolism was positively correlated with Testosterone Biosynthesis Process which was consistent with previous findings, implying that efficient pyruvate metabolism may also play an important role in testosterone synthesis.

In Control and COVID-19 groups, Wp Cholesterol Metabolism Pathways, Wp Cholesterol Biosynthesis Pathway, Go Testosterone Biosynthesis Process. KEGG Glycolysis Gluconeogenesis, Wp Glycolysis And Gluconeogenesis, Wp Glycolysis In Senescence, Wp Estrogen Metabolism were highly positively correlated with pyruvate metabolism (Figures 5G, H), suggesting that normal glycolysis plays a key role in pyruvate synthesis. Testosterone Biosynthesis Process was also significantly positively correlated with the normal pyruvate synthesis. On the other hand, Go Germ Cell Proliferation, KEGG MAPK Signaling Pathway, KEGG P53 Signaling Pathway, Wp Activation Of Nlrp3 Inflammasome By Sarscov2, Wp Ras And Bradykinin Pathways In COVID-19, Wp Ras Signaling, Wp Complement Activation, Wp Human Complement System, Go Senescence were negatively correlated with pyruvate metabolism in both Control and COVID-19 groups (Figures 5G, H), which proved that cell cycle damage was significantly negatively correlated with normal pyruvate metabolism. Go senescence was found to be strongly inversely associated to normal pyruvate metabolism. Multiple signaling pathways were altered as a result of COVID-19 infection. The Go Spermatid Differentiation Pathway was positively correlated with pyruvate metabolism (Figures 5G, H) in the control group, while negatively correlated with pyruvate metabolism in the COVID-19 group, indicating that sperm differentiation was hindered following COVID-19 infection.

Given the negative correlation between cellular senescence and energy metabolism in the testis, particularly glycolysis, it is critical to understand the internal mechanism between cellular senescence and glycolysis. To learn more about how cellular senescence affects the glycolytic process during COVID-19 infection, we evaluated important genes that are positively and negatively related with glycolytic rate-limiting enzymes (HK, PLKR, PRM). Firstly, 637 genes with strong negative correlation with HK1 (*r* < −.9, *p* < .05) were detected by PPI on STRING website, and the top 20 genes were obtained (Figure 5I). At the same time, PPI was applied to 415 genes that with strong positive correlation with HK1(*r* > .9, *p* < .05) in order to produce the top 20 ones (Figure 5J). The positive and negative genes correlated with the remaining core rate-limiting enzyme were treated by using the same manner (S1).

In the relevant literatures, 13 genes have been proven to have effects on cellular senescence. Interestingly, MAPK1 is the most obvious gene that has a negative relationship with HK1(*r* = −1.0, *p* < .001) (Figure 5K). This gene is implicated in the COVID-19-induced cell senescence signaling pathway. COVID-19-induced cell senescence is also expected to impede testis glycolysis through this gene. As a result, MAPK1 could become a key link between COVID-19, cellular senescence, and glycolysis.

6) MAPK pathway plays an important role in COVID-19 infection inhibiting spermatogenesis. Targeted MAPK pathway drugs are expected to effectively inhibit the damage of COVID-19 to spermatogenesis.

COVID-19 induces testicular cell senescence primarily through extracellular regulated protein kinases (ERK) and p38 mitogen-activated protein kinase (p38 MAPK) in four subfamilies of the MAPK signaling pathway, as shown in the previous analysis (Figure 2E). Through Figure 2F, we can show that ERK (MAPK1) protein and P38 (MAPK14) protein play a key role in inducing cellular senescence by COVID-19. In correlation analysis, MAPK pathway and cellular senescence were both negatively correlated with testosterone synthesis and glycolysis (Figures 3F, G). At the same time, MAPK1 shows the strongest negative connection with glycolysis’s primary rate-limiting enzyme, HK1, indicating that it may efficiently inhibit glycolysis. As a result, the MAPK pathway may play an essential role in COVID-19 inducing cellular senescence and inhibiting spermatogenesis.

**FIGURE 6:**
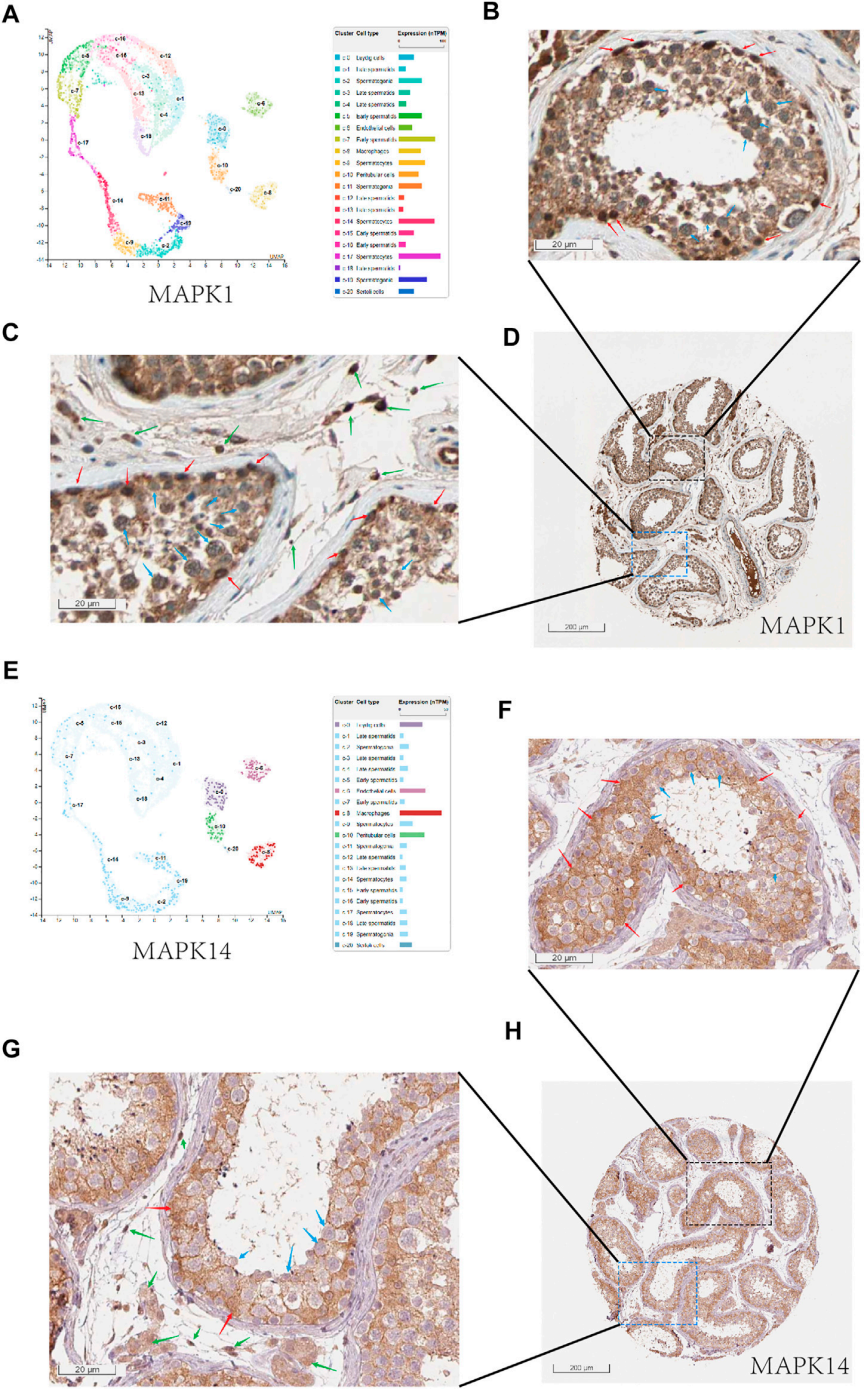
The expression of MAPK1 and MAPK14 in spermatogenic tubules. **(A)** Through the single cell analysis of HPA online website, it can be seen that MAPK1 is mainly expressed in sertoli cells, leydig cells, and spermatogenic cells in the human normal testis.**(B)** Through the human protein expression analysis website of HPA online website, it can be seen that MAPK1 is mainly expressed in sertoli cells (red arrow) and spermatogenic cells (blue arrow) in the human normal testis (400x).**(C)** and it can be seen that MAPK1 is mainly expressed in leydig cells (green arrow) and spermatogenic cells (blue arrow) in the human normal testis (400x).**(D)** Overall expression of MAPK1 protein in seminiferous tubules (10x). **(E)** Through the single cell analysis of HPA online website, it can be seen that MAPK14 is mainly expressed in leydig cells and sertoli cells in the human normal testis. **(F)** Through the human protein expression analysis website of HPA online website, it can be seen that MAPK14 is mainly expressed in sertoli cells (red arrow) in the human normal testis (400x). **(F)** and it can be seen that MAPK14 is mainly expressed in leydig cells (green arrow) in the human normal testis (400x). **(G)** Overall expression of MAPK14 protein in seminiferous tubules (10x). **(H)** Overall view of the MAPK14 expression of human normal testis slice.

In order to effectively reduce the inhibition of COVID-19 on spermatogenesis, we hope to find drugs that can effectively inhibit the negative effect of MAPK pathway in COVID-19 infected patients, especially for MAPK1 and MAPK14. Firstly, we looked at the locations of MAPK1 and MAPK14 in human normal testis cells. We can see that mRNA-MAPK1 is significantly expressed in Sertoli cells, Leydig cells, and spermatogenic cells in the human normal testis single-cell analysis by using HPA (https://www.proteinatlas.org/) (Figure 6A). At the same time, we use HPA to evaluate the MAPK1 protein expression on the human protein expression analysis website. MAPK1 is also mostly found in Sertoli cells, Leydig cells, and spermatogenic cells (Figures 6B–D), which is consistent with single-cell analysis results. In single-cell analysis, MAPK14 was primarily found in Leydig cells and Sertoli cells, endothelial cells, and macrophages (Figure 6E), and in the human protein expression analysis website of HPA, it was primarily found in Leydig cells and Sertoli cells (Figures 6F–H). In conclusion, MAPK1 and MAPK14 were found to be highly expressed in both Leydig cells and Sertoli cells in human normal testis cells. This laid a molecular foundation for COVID-19 to induce cellular senescence through MAPK signaling pathway, thereby inhibiting androgen synthesis and glycolysis process.

From the previous results, we can see that MAPK1 and MAPK14 play an important role in COVID-19 infection on cellular senescence and inhibition of androgen synthesis and reducing glycolysis. In combination with the recommendations of Chinese patent medicines in the current new coronavirus treatment guidelines, we found that Qingfei Paidu Decoction, Jinhua Qinggan Granule, Reduning Injection, Tanreqing Injection and Shufeng Jiedu Capsules all played certain therapeutic effects on MAPK1 and MAPK14 targets in patients with COVID-19 infection after consulting a large number of literatures. Interestingly, the majority of these patented Chinese medications target multiple MAPK enzymes in the MAPK pathway, including the MAPK1, MAPK8, MAPK14 (Table 1).

**TABLE 1:** Study on the mechanism of action of traditional Chinese medicine recommended by China COVID-19 guidelines for diagnosis and treatment targeting MAPK1/MAPK14.

Chinese medicine	Constituent	Predicted active ingredient	Predicted target	Signaling pathway and mechanism		References
				Anti- inflammation and immunoregulation	Target organs protection	
Qingfei Paidu decoction	21 herbs, including *Pogostemonis Herba, Glycyrrhizae Radix et Rhizoma, Ephedrae Herba, Armeniacae Semen Amarum, Scutellariae Radix, Asteris Radix et Rhizoma,* and *Polyporus,* etc.	Quercetin, luteolin, kaempferol, beta-sitosterol, naringenin, isorhamnetin, patchouli alcohol, ergosterol, shionone, tussilagone, 3,4-dicaffeoylquinic acid, 4,5-dicaffeoylquinic acid, baicalein, glycyrrhizic acid, etc.	MAPK8, IL-6, COX-2, mPGES-1, AKT1, **MAPK1**, JUN, TNF, EGFR, CASP3, IL1B, PTGS2, NOS2, **MAPK14**, etc.	Arachidonic acid metabolism pathway, TNF, NF-κB, MAPK, non-small cell lung cancer, small cell lung cancer, IL17, tuberculosis, Th17, pertussis signal pathway, TLR, etc.	TNF, NF-κB signaling pathways, PI3K-Akt pathway, MAPK pathway, T cell pathway, B cell pathway, Ras, apoptosis, etc.	Ren et al. (2020), Wu et al. (2020), Zhao et al. (2020), Xu, et al. (2020), Wang et al. (2020), Ruan et al. (2020), Fan, et al., Zhang et al. (2004)
						Yang et al. (2020) (Chemical composition and pharmacological mechanism of Qingfei Paidu Decoction and Ma Xing Shi Gan Decoction against Coronavirus Disease 2019 (COVID-19): In silico and experimental study - PMC)
Jinhua Qinggan granules	21 herbs, including *Pogostemonis Herba, Glycyrrhizae Radix et Rhizoma, Ephedrae Herba, Armeniacae Semen Amarum, Scutellariae Radix, Asteris Radix et Rhizoma,* and *Polyporus,* etc.	Quercetin, luteolin, kaempferol, beta-sitosterol, naringenin, isorhamnetin, patchouli alcohol, ergosterol, shionone, tussilagone, 3,4-dicaffeoylquinic acid, 4,5-dicaffeoylquinic acid, baicalein, glycyrrhizic acid*,* etc.	MAPK8, IL-6, COX-2, mPGES-1, AKT1, **MAPK1**, JUN, TNF, EGFR, CASP3, IL1B, PTGS2, NOS2, **MAPK14**, etc.	Arachidonic acid metabolism pathway, TNF, NF-κB, MAPK, non-small cell lung cancer, small cell lung cancer, IL17, tuberculosis, Th17, pertussis signal pathway, TLR, etc.	TNF, NF-κB signaling pathways, PI3K-Akt pathway, MAPK pathway, T cell pathway, B cell pathway, Ras, apoptosis, etc.	Simayi et al. (2020) Jimilihan et al. (2020), Ren et al. (2020) Shen, et al. Liu et al. (2015), Mao et al. (2020b)
Reduning injection	3 herbs, including *Lonicerae Japonicae Flos, Artemisiae Annuae Herba,* and *Gardeniae Fructus*	Quercetin, luteolin, lonicerin, isorhamnetin, salicylic acid	COX-2, sEH, IL6, CCL2, CASP3, IL4, **MAPK1**, RELA, FOS, NOS2, IL1B, CXCL10, **MAPK14**, EGFR etc.	Arachidonic acid metabolism pathway, IL-17 signaling pathway, C-type lectin receptor signaling pathway, HIF-1 signaling pathway and NF-*κ*B signaling pathway etc.	IL-17 signaling pathway	Ren et al. (2020), Sun et al. (2020)
Tanreqing injection	5 herbs, including *Lonicerae Japonicae Flos, Forsythiae Fructus,* and *Scutellariae Radix,* etc.	Lonicerin, quercetin, kaempferol, luteolin, baicalein, wogonin etc.	COX-2, sEH, LTA4H, IL6, IL1B, IL10, **MAPK1**, IL4, CXCL8, **MAPK14**, EGFR, CXCL10 etc.	Arachidonic acid metabolism pathway, Th 17 cell differentiation, MAPK, EGFR, TNF signal pathway, etc.		Ren et al. (2020), Kong (2020)
Shufeng Jiedu capsule	8 herbs, including *Polygoni Cuspidati Rhizoma et Radix, Forsythiae Fructus, Isatidis Radix,Bupleuri Radix, Patrinia Herb, Verbenae Herb, Phragmits Rhizoma, Glycyrrhizae Radix et Rhizoma*	Quercetin, luteolin, wogonin, kaempferol, β-sitosterol, acacetin, puerarin, licochalcone A, isorhamnetin, 5,7,4′-trihydroxy-8-methoxyflavone, β-sitosterol, 6-(3-oxoindolin-2-ylidene) indolo [2,1-b] quinazolin-12-one, bicuculline, licoisoflavanone etc.	IL6, IL1B, CCL2, IL2, MAPK8, **MAPK1**, **MAPK14**, CASP3, FOS, ALB, IL4, IL1B, EGFR, FOS, AR, BCL2L, NOS2, F10, PTGS2, PTGS1, ESR1, DPP4 etc.	HIF-1, NF-*κ*B, IL-17, EGFR, MAPK, endocrine resistance, tyrosine kinase resistance, platinum drug resistance, antifolate resistance, arginine biosynthesis, sphingolipid, human cytomegalovirus infection, Kaposi’s sarcoma, small cell lung cancer etc.	Endocrine resistance	Xu et al. Sun et al. (2020), Shen Q. et al. (2020), Cao, (2020)

## Discussion

COVID-19 has been linked to male spermatogenesis dysfunction in numerous previous studies, however the exact mechanism is still unknown. ACE2, as an essential role in COVID-19 infected testis, mainly expressed in Sertoli cells and Leydig cells. This study speculaed that COVID-19 can induce testicular cell senescence through MAPK signaling pathway, and cellular senescence negatively correlated with glycolysis metabolism, normal pyruvate and lactate metabolism mainly performed in Sertoli cells, testosterone synthesis and cholesterol synthesis mainly performed in Leydig cells. Drugs target on MAPK signaling pathway, especially on MAPK1, MAPK14, are expected to become effective therapeutic drugs to reduce the induction of cell senescence by COVID-19.

Cellular senescence is a special state of cells, which can permanently stop the cell cycle (Muñoz-Espín and Serrano, 2014), and at the same time promote the appearance of morphological changes such as the flattening and expansion of nucleus and nucleolus, and the appearance of vacuoles in cytoplasm (Hernandez-Segura et al., 2018). Cellular senescence can be induced by exposure to excessive external stimuli. These stimuli include activated oncogenes, DNA damage agents, oxidative stress, destruction of epigenetic regulation and ectopic expression of tumor suppressors (Muñoz-Espín and Serrano, 2014; Hernandez-Segura et al., 2018). Virus invasion reflects a major cell damage and induces significant biological changes in the infected host cells, which may be used as a state transition caused by viruses to lead to cellular senescence (Martínez et al., 2016). After COVID-19 virus infection in humans, cellular senescence can be promoted by inflammatory factors, oxidative stress, complement activation and DNA damage (Chandrasekaran et al., 2016; Coperchini et al., 2020). In this paper, the differential genes enrichment analysis of testicular mRNA in patients with COVID-19 infection showed that COVID-19 induces many cell cycle arrest pathways, in which the cellular senescence pathway was the most obvious. So we speculated that COVID-19 infection induced testicular cell senescence. This is consistent with the previous studies that COVID-19 can cause cellular senescence (Nehme et al., 2020).

To further understand how COVID-19 induces cellular senescence, we analyzed the correlation between cellular senescence and other pathways, and found that MAPK pathway was most positively correlated with cellular senescence. MAPK pathway can be activated by all kinds of inflammatory factors, products of oxidative stress. In particular, ERK and P38-MAPK in the MAPK pathway are enriched in the process of cell senescence induced by inflammatory factors, oxidative stress and other factors in this study (Figure 2E). IL1-β, MCP-1, IL10 and TGF-αin the testis increased significantly after COVID-19 infection according to the studies by Kharbach and Khallouk (2021). These components can effectively activate MAPK pathway. A large number of factors can be secreted by SASP related to cellular senescence, including IL-6, IL-8, CCL2 and CXCL1, plasminogen activator inhibitor 1 (PAI-1), IGFBP7 and TGF-b (Coppé et al., 2008; Kuilman et al., 2008; Chemokine signaling *via* the CXCR2 receptor reinforces senescence—PubMed). These components enhance the MAPK signaling pathway in turn, aggravating cellular senescence, resulting in a vicious loop that worsens the spermatogenesis damage induced by cellular senescence. In conclusion, it can see that MAPK pathway was activated by inflammation induced by COVID-19, which further aggravated testicular cell senescence.

Previous research has found that COVID-19-induced senescence exacerbates lung injury, lung failure, cardiac dysfunction, nervous system infection, and gut injury (Oudit et al., 2009; Chu et al., 2020; Mao et al., 2020a; Mason, 2020, 19). COVID-19 has also been identified in recent study to have a negative impact on infertile men’s sexual function, perhaps resulting in sperm quality decline (Ma et al., 2020; Gul et al., 2021; Koç and Keseroğlu, 2021). COVID-19-induced inflammatory substances also impact sperm quality by impairing the blood testosterone barrier (Peirouvi et al., 2021). Prior study has demonstrated that COVID-19 has a negative impact on spermatogenesis, but the underlying mechanism has yet to be completely investigated.

COVID-19 has been linked to spermatogenesis damage in prior studies (Gul et al., 2021; Kharbach and Khallouk, 2021; Koç and Keseroğlu, 2021). According to the results of a biopsy of the testis of COVID-19-infected dead patients, many spermatogenic tubules in the infected testis have almost no complete spermatogenic cells, while the number of Sertoli cells in the infected patients’ testis and the non-infected controls’ testis is comparable (Ma et al., 2020). Another study found that after treatment for COVID-19, the semen volume, total motile rate, progressive motile rate, and normal sperm morphology were all reduced significantly (Koç and Keseroğlu, 2021). The COVID-19 may have an adverse effect on male infertility patients’ gonadal function, leading to a worsening of semen parameters (Koç and Keseroğlu, 2021). COVID-19-induced inflammatory factors may disrupt the blood-testis barrier, altering the spermatogenesis according to Peirouvi et al. (2021). Although prior research has shown that COVID-19 has a deleterious impact on spermatogenesis, the underlying mechanism has yet to be fully investigated.

To learn more about how COVID-19 inhibits spermatogenesis, particularly cellular senescence, the disease’s principal pathological change. We discovered that testicular cell senescence negatively correlated with glycolysis pathway, normal pyruvate metabolism, testosterone synthesis pathway and cholesterol synthesis pathway.

Androgen is a kind of steroid compound, mainly produced by endoplasmic reticulum in testicular Leydig cells (Smith and Walker, 2014). Testosterone is the most important member of the androgen family, as well as the most important hormone to initiate and maintain spermatogenesis (Smith and Walker, 2014). Androgen *in vivo* is mainly converted from cholesterol through a series of biochemical processes (Miller, 2002). Adequate cholesterol metabolism is critical for normal androgen synthesis in the testis. Testosterone levels in patients with COVID-19 have been reported to be considerably lower in previous studies. Most patients with COVID-19 have testosterone insufficiency, and severe patients’ testosterone levels are much lower (Okçelik, 2021; Salonia et al., 2021).

In order to explore how COVID-19 reduces the level of androgen, thereby affecting the spermatogenesis. In COVID-19 group, we examined at the positive and negative correlation genes and pathways of the androgen synthesis pathway, and discovered that the pathways negatively correlated with androgen synthesis were mostly cell cycle inhibition related pathways, especially cellular senescence. Previous research has revealed that cellular senescence can damage the endoplasmic reticulum, disrupting its homeostasis and impairing its normal function (Pluquet and Abbadie, 2021). The endoplasmic reticulum is where steroid hormones are synthesized in cells. Damage to the endoplasmic reticulum in Leydig cells caused by COVID-19-induced senescence is bound to disrupt testosterone synthesis. Therefore, we speculate that Leydig cells may undergo cellular senescence after COVID-19 infection, affecting the androgen synthesis function of testicular Leydig cells. SRSF1, NIPBL, the hub genes negatively correlated with essential enzymes in androgen synthesis, have been implicated in the process of cellular senescence in earlier research (Blanco and Bernabéu, 2012; Gu et al., 2021), demonstrating the importance of cellular senescence in COVID-19-induced androgen synthesis suppression.

Total ATP required for primary spermatocyte meiosis and spermatocyte depends on total lactate and pyruvate production by Sertoli cells during spermatogenesis (Boussouar and Benahmed, 2004; Rato et al., 2012). In the spermatogenesis, the energy—lactate and pyruvate—is created by the efficient glycolysis of Sertoli cells under the action of FSH (Jutte et al., 1981). Glycolysis has been demonstrated to be inhibited by cellular senescence in previous research. Such as Stephen C’s research has found that brain’s aerobic glycolysis was harmed due to cellular senescence (Cunnane et al., 2020).

The mechanism of how cellular senescence impair testicular cell metabolism has not been fully understood in previous studies. It is well known that whether glycolysis go on well is mainly determined by three key enzymes, including HK, PRLR and RPL (Tan and Miyamoto, 2015; Huang et al., 2021; Nie et al., 2021). We hypothesized that the glycolysis level fell dramatically after COVID-19 infection because these three key enzymes decreased significantly. The ssGSEA of the glycolysis between the two groups showed the same results, as expected. In order to learn more about how COVID-19 inhibits glycolysis after COVID-19 infection, we found that MAPK signaling pathway and cellular senescence induced by COVID-19 were significantly negatively correlated with glycolysis through the correlation analysis of glycolysis. At the same time, we ran the protein interaction network analysis for the negative correlation genes of the three key enzymes in glycolysis. The results showed that in the top 20 hub genes negatively correlated with the three enzymes respectively, many genes had been reported to be related to cellular senescence, including MAPK1, MITF, JUP, COX5 A, RPL22, RPLP1, DNA2, RPL31 and LRRK2 (Wang et al., 2016; Zhou et al., 2022). Interestingly, among these senescence-related genes, MAPK1 occupied the most core position of the key rate-limiting enzyme HK1 negative correlation genes in glycolysis, which proves that MAPK signaling pathway, especially MAPK1, plays an important core function in inhibiting glycolysis. Combined with the statistical result that cellular senescence could inhibit glycolysis by Stephen C’s research. We speculated that COVID-19-induced cellular senescence in combination with MAPK signaling pathway plays an important role in inhibiting glycolysis, thereby inhibiting the synthesis of pyruvate and lactic acid, reducing the energy required for spermatogenesis, and ultimately affecting the spermatogenesis. This is a new insight into how spermatogenesis inhibited by COVID-19.

COVID-19’s cell receptor is angiotensin-converting enzyme 2 (Verdecchia et al., 2020). According to previous studies on the relationship between angiotensin-converting enzyme and COVID-19, angiotensin-converting enzyme is essential in COVID-19 infected testis (Shen Q et al., 2020). In the testis, ACE2 is mostly expressed in Sertoli cells and Leydig cells, whose main function is to provide energy and androgen for spermatogenesis (Boussouar and Benahmed, 2004; Douglas et al., 2004; Shen F et al., 2020). This study found that the senescence induced by COVID-19 was negatively correlated with androgen synthesis pathway and glycolysis process. As a result, we hypothesized that COVID-19 infection mostly accelerated the senescence of testicular cells, particularly Sertoli cells and Leydig cells, disrupting spermatogenesis. The manifestations of these two cell senescence were consistent with the findings of Yang M et al. (2020), that the Sertoli cells showed swelling, vacuolation, cytoplasmic rarefaction, and the Leydig cells showed swelling in the testicular specimens of 12 patients with COVID-19 after death.

**FIGURE 7:**
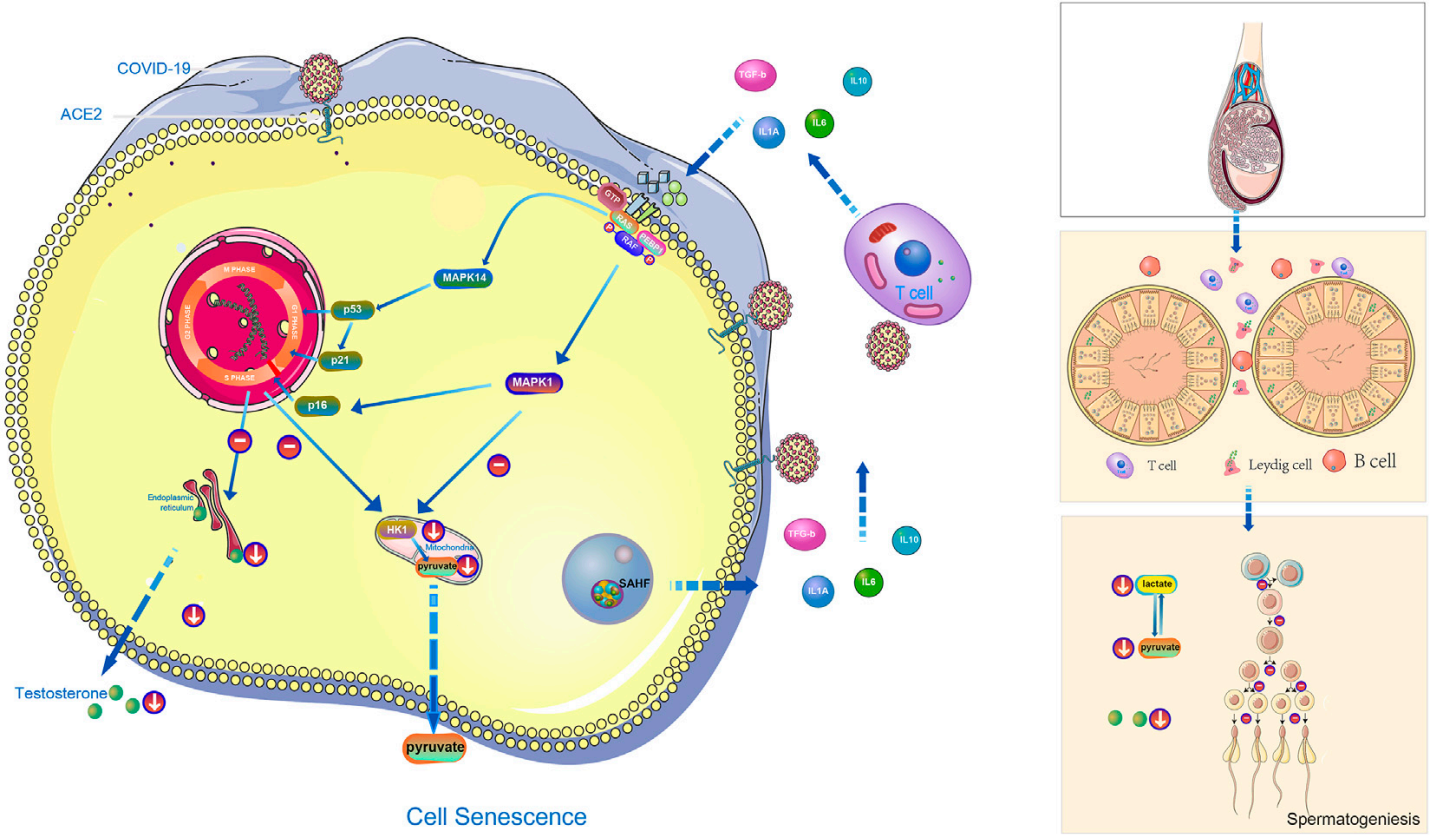
The hypothetical diagram of the results in this study. In COVID-19 infection,COVID-19 can induce testicular cell senescence through MAPK signaling pathway (upper right and middle position of left half figure), and cellular senescence synergize with MAPK pathway to further affects the normal synthesis of cholesterol and androgen (lower right and middle position of left half figure), and to inhibits normal synthesis of lactate and pyruvate (Right below the left half figure), ultimately affects male spermatogenesis (Right half diagram, from top to bottom is the structure of testis, seminiferous tubules and their surrounding environment, spermatogenesis process).

MAPK signaling pathway mainly includes extracellular-signal regulated protein kinase (ERK) , p38 mitogen activated protein kinase (p38 MAPK), c-Jun N-terminal kinase (JNK) , extracellular signal regulated kinase 5 (ERK5) (Kim and Choi, 2010). Peirouvi’s study showed severe “inflammatory storm” in the testis after COVID-19 infection (Peirouvi et al., 2021). Our study showed that COVID-19 promoted testicular cell senescence (Figure 2E) mainly through extracellular-signal regulated protein kinase (ERK) and p38 mitogen-activated protein kinase (p38 MAPK) under the action of multiple factors such as inflammatory storm. Cellular senescence in the coordination of MAPK signaling pathway has a significant inhibitory effect on androgen synthesis and cholesterol synthesis, also glycolysis and pyruvate metabolism, and ultimately affects the spermatogenesis (Figure 7). This provides a blueprint of complete mechanism for how COVID-19 inhibits spermatogenesis.

As an important member of the MAPK pathway, MAPK enzymes are a group of evolutionary conserved serine, threonine protein kinases, which are activated by a series of extracellular stimuli including peptide growth factors, cytokines, hormones and various cell stressors, such as oxidative stress and endoplasmic reticulum stress, and ultimately mediate signal transduction from the cell membrane to the nucleus. They control a variety of cell functions, including inflammation, proliferation, differentiation, survival, cancer, and death (Kim and Choi, 2010). Our study showed that ERK (MAPK1 protein) and P38 (MAPK14 protein) played important roles in mediating COVID-19 to induce cellular senescence through MAPK pathway. Meanwhile, MAPK1 played a core function in the process of COVID-19 induced cellular senescence inhibiting glycolysis. In a previous study, Ni can inhibit testosterone synthesis by activating ERK1/2 (MAPK1) and p38MAPK (MAPK14) signaling pathways in Leydig cells. In summary, we speculated that MAPK1 and MAPK14 may function as a bridge among COVID-19 infection, cellular senescence, inhibition of androgen synthesis and inhibition of glycolysis. This study provides molecular targets for exploring how to effectively reduce the inhibitory effect of COVID-19 on spermatogenesis.

Activation of p38 (MAPK14) -MAPK pathway promotes inflammation, vasoconstriction and thrombosis, and contributes to viral life cycle (Zarubin and Han, 2005; Marchant et al., 2010). Previous studies have shown that inhibition of p38 (MAPK14) -MAPK pathway can effectively alleviate the symptoms of COVID-19 infection, which is a promising treatment for COVID-19 (Grimes and Grimes, 2020). In order to effectively lower the inhibition of COVID-19 on spermatogenesis, medications that can effectively inhibit the MAPK pathway in COVID-19 infected patients were seek, especially MAPK1 and MAPK14. We found that Qingfei Paidu Decoction, Jinhua Qinggan Granule, Reduning Injection, Tanreqing Injection and Shufeng Jiedu Capsules all played certain therapeutic effects on MAPK1 and MAPK14 targets in patients with COVID-19 infection . This study provides effective medications for how to reduce COVID-19 inhibition of spermatogenesis in the future.

## Conclusion

In summary, this study found that COVID-19 could induce testicular cell senescence through MAPK signaling pathway. Cellular senescence in the coordination of MAPK signaling pathway, which further affected the normal synthesis of cholesterol and androgen, inhibited the normal synthesis of lactate and pyruvate, and ultimately affected spermatogenesis. The medications targeting MAPK signaling pathway, especially MAPK1 and MAPK14, are expected to be effective therapeutic medications for reducing COVID-19 damage to spermatogenesis. These result give us a new insight into how COVID-19 inhibits spermatogenesis and provide a possible solution to mitigate this damage.

## Data Availability

The datasets presented in this study can be found in online repositories. The names of the repository/repositories and accession number(s) can be found in the article/Supplementary Material.

## Supplementary material

SUPPLEMENTARY FIGURE S1
**(A)** The top 20 genes strong negative correlation with HK2 (*r* < −.9, *p* < .05) by PPI and the top 20 genes strong positive correlation with HK2 (*r* > .9, *p* < .05) by PPI. **(B)** The top 20 genes strong negative correlation with HK3 (*r* < −.9, *p* < .05) by PPI and the top 20 genes strong positive correlation with HK3 (*r* > .9, *p* < .05) by PPI. **(C)** The top 20 genes strong negative correlation with PFKM (*r* < −.9, *p* < .05) by PPI and the top 20 genes strong positive correlation with PFKM (*r* > .9, *p* < .05) by PPI. **(D)** The top 20 genes strong negative correlation with PFKR (*r* < −.9, *p* < .05) by PPI and the top 20 genes strong positive correlation with PFKR (*r* > .9, *p* < .05) by PPI. **(E)** The top 20 genes strong negative correlation with PKLR (*r* < −.9, *p* < .05) by PPI and the top 20 genes strong positive correlation with PKLR (*r* > .9, *p* < .05) by PPI. **(F)** The top 20 genes strong negative correlation with PKM (*r* < −.9, *p* < .05) by PPI and the top 20 genes strong positive correlation with PKM (*r* > .9, *p* < .05) by PPI.

SUPPLEMENTARY FIGURE S2 After WIKIPATHWAYS enrichment of genes significantly decreased in patients with COVID-19 infection, the first 10 pathways with the most significant p value were obtained.

SUPPLEMENTARY TABLE S1 Genes contained in related pathways (GOBP_CELLULAR_SENESCENCE, KEGG_SENESCENCE, KEGG Coronavirus_disease COVID-19).
