# Supplementary material for: COVID-19 inhibits spermatogenesis in the testes by inducing cellular senescence
Source: Front Genet. 2023 Jan 5;13:981471. doi: 10.3389/fgene.2022.981471 (PMC9849386; doi:10.3389/fgene.2022.981471)
Supplement: Supplementary file 1 [file Table1.DOCX]

| Pathway | Genes |
| --- | --- |
| GOBP_CELLULAR_SENESCENCE  (GO_SENESCENCE) | ABL1 AKT3 AKT3 ARG2 ARNTL B2M B2M BCL2L12 BCL6 BMPR1A CALR CDK6 CDKN1A CDKN2A CDKN2B CGAS ECRG4 EEF1E1 FBXO5 H2AX HLA-G HLA-G HLA-G HLA-G HLA-G HLA-G HLA-G HLA-G HMGA1 HMGA2 HRAS HRAS ID2 ING2 KAT6A KIR2DL4 KIR2DL4 KIR2DL4 KIR2DL4 KIR2DL4 KIR2DL4 KIR2DL4 KIR2DL4 KIR2DL4 KIR2DL4 KIR2DL4 KIR2DL4 KIR2DL4 KIR2DL4 KIR2DL4 KIR2DL4 KIR2DL4 KIR2DL4 KIR2DL4 KIR2DL4 KIR2DL4 KIR2DL4 KIR2DL4 KIR2DL4 KIR2DL4 KIR2DL4 KIR2DL4 KIR2DL4 KIR2DL4 KIR2DL4 KIR2DL4 KIR2DL4 KIR2DL4 KIR2DL4 KIR2DL4 KIR2DL4 KIR2DL4 KIR2DL4 KIR2DL4 KIR2DL4 KIR2DL4 KRAS MAGEA2 MAGEA2B MAP2K1 MAP3K3 MAPK14 MAPKAPK5 MIR10A MIR146A MIR17 MIR188 MIR20B MIR217 MIR22 MIR34A MIR543 MIR590 NEK4 NEK6 NSMCE2 NUAK1 OPA1 PAWR PLA2R1 PLK2 PML PNPT1 PRKCD PRKDC PRMT6 RBL1 RSL1D1 SIRT1 SLC30A10 SMC5 SMC6 SPI1 SRF TBX2 TBX3 TERC TERF2 TERT TP53 TWIST1 ULK3 VASH1 WNT16 YBX1 YPEL3 ZKSCAN3 ZMPSTE24 ZNF277 |
| KEGG_SENESCENCE | MAPKAPK2 CDC25A CDKN2B MAPK13 FOXM1 CCNE1 MAPK12 CALM3 MTOR PIK3R2 TSC2 HRAS MYBL2 ITPR2 MAP2K2 CCNA1 HLA-G HIPK4 |
| KEGG Coronavirus_disease COVID-19 | TMPRSS2 ACE2 NRP1 ACE MAS1 AGTR1 CYBB NFKB1 RELA TNF IL6 IL1B IL12A IL12B MMP3 MMP1 CCL2 CXCL8 ADAM17 TNFRSF1A NFKBIA NFKBIB HBEGF EGFR IL6R IL6ST JAK1 TYK2 STAT3 IL2 CSF3 CSF2 CXCL10 RPS2 RPS3 RPS3A RPS4Y1 RPS4X RPS4Y2 RPS5 RPS6 RPS7 RPS8 RPS9 RPS10 RPS10-NUDT3 RPS11 RPS12 RPS13 RPS14 RPS15 RPS15A RPS16 RPS17 RPS18 RPS19 RPS20 RPS21 RPS23 RPS24 RPS25 RPS26 RPS27 RPS27L RPS27A RPS28 RPS29 FAU RPSA RPL3L RPL3 RPL4 RPL5 RPL6 RPL7 RPL7A RPL8 RPL9 RPL10L RPL10 RPL10A RPL11 RPL12 RPL13 RPL13A RPL14 RPL15 RPL17 RPL17-C18orf32 RPL18 RPL18A RPL19 RPL21 RPL22L1 RPL22 RPL23 RPL23A RSL24D1 RPL24 RPL26 RPL26L1 RPL27 RPL27A RPL28 RPL29 RPL30 RPL31 RPL32 RPL34 RPL35 RPL35A RPL36 RPL37 RPL37A RPL38 RPL39 UBA52 RPL41 RPL36AL RPL36A RPL36A-HNRNPH2 RPLP0 RPLP1 RPLP2 IGH FCGR2A SYK PIK3CA PIK3CD PIK3CB PIK3R1 PIK3R2 PIK3R3 PLCG1 PLCG2 PRKCA PRKCB PRKCG MAPK1 MAPK3 MAPK11 MAPK12 MAPK13 MAPK14 TLR2 TLR4 MYD88 TLR7 TLR8 IRAK4 IRAK1 TRAF6 MAP3K7 TAB2 MAPK8 MAPK10 MAPK9 FOS JUN CHUK IKBKB IKBKG NLRP3 CASP1 TLR3 DDX58 ISG15 IFIH1 MAVS TRAF3 IKBKE TBK1 IRF3 CGAS STING1 IFNA1 IFNA2 IFNA4 IFNA5 IFNA6 IFNA7 IFNA8 IFNA10 IFNA13 IFNA14 IFNA16 IFNA17 IFNA21 IFNB1 IFNAR1 IFNAR2 STAT1 STAT2 IRF9 ADAR OAS1 OAS2 OAS3 MX1 MX2 EIF2AK2 C5AR1 C3AR1 C3 CFB CFD C5 C6 C7 C8A C8B C8G C9 SELP VWF C1QA C1QB C1QC C1R C1S C2 C4A C4B MBL2 MASP1 MASP2 F2 F13A1 F13B FGA FGB FGG |
| GO_SPERMATID_DIFFERENTIATION | ABHD2 ACRBP ADAD1 AFF4 ARMC2 BAX BBS2 BBS4 BRIP1 BSPH1 CABYR CAPZA3 CATSPER2 CATSPER3 CATSPER4 CATSPERD CATSPERZ CCDC136 CCDC42 CCDC63 CCR6 CDYL CEP131 CEP57 CFAP157 CFAP206 CFAP43 CFAP44 CFAP65 CFAP69 CFAP97D1 CFTR CHD5 CIB1 DDX25 DDX6 DEFB1 DEFB1 DHH DLD DMC1 DNAH1 DPY19L2 DPY19L2P1 DPY19L2P2 DPY19L2P2 DYNLL1 EFCAB9 ELSPBP1 FABP9 FAM9A FAM9B FAM9C FANCG FSCN3 FSIP2 GALNTL5 H1-7 H2BC1 H3-3A H3-3B HMGB2 HOOK1 HSPA2 ING2 IQCF1 IQCG JAM2 JAM3 KDM3A KIT KLHL10 KNL1 MAST2 MEIG1 MEIOC MKKS NECTIN2 NECTIN3 NEURL1 NME5 NPHP1 NSUN2 OCA2 OCA2 ODF2 OSBP2 PACRG PAEP PAFAH1B1 PANK2 PCSK4 PDILT PITHD1 PIWIL1 PIWIL1 PLA2G3 PLD6 PRKACA PRKACA PRM2 PSME4 PTCHD3 PTCHD3 PYGO1 PYGO2 QKI REC8 REC8 RFX2 RHBDD1 RIMBP3 RIMBP3B RIMBP3C RNF8 ROPN1 ROPN1B ROPN1L RSPH1 RSPH6A SEMG1 SEMG2 SIX5 SLC26A3 SLC26A6 SLC26A8 SMARCA2 SOX30 SPACA1 SPAG16 SPANXB1 SPEF2 SPEM1 SPEM1 SPINK1 SPINK2 SPO11 SRPK1 STRBP SUN5 SYCP1 SYCP3 TARBP2 TBPL1 TCP11 TCP11X1 TDRD5 TMEM119 TMF1 TNP1 TNP2 TOPAZ1 TPGS1 TRIP13 TSSK1B TSSK2 TSSK4 TSSK4 TSSK6 TTC12 TTC21A TTC26 TTLL1 TXNDC8 UBE2B UBE2J1 YTHDC2 ZMYND15 ZPBP ZPBP2 |
|  |  |
