# Supplementary figures and images for: COVID-19 inhibits spermatogenesis in the testes by inducing cellular senescence
Source: Front Genet. 2023 Jan 5;13:981471. doi: 10.3389/fgene.2022.981471 (PMC9849386; doi:10.3389/fgene.2022.981471)

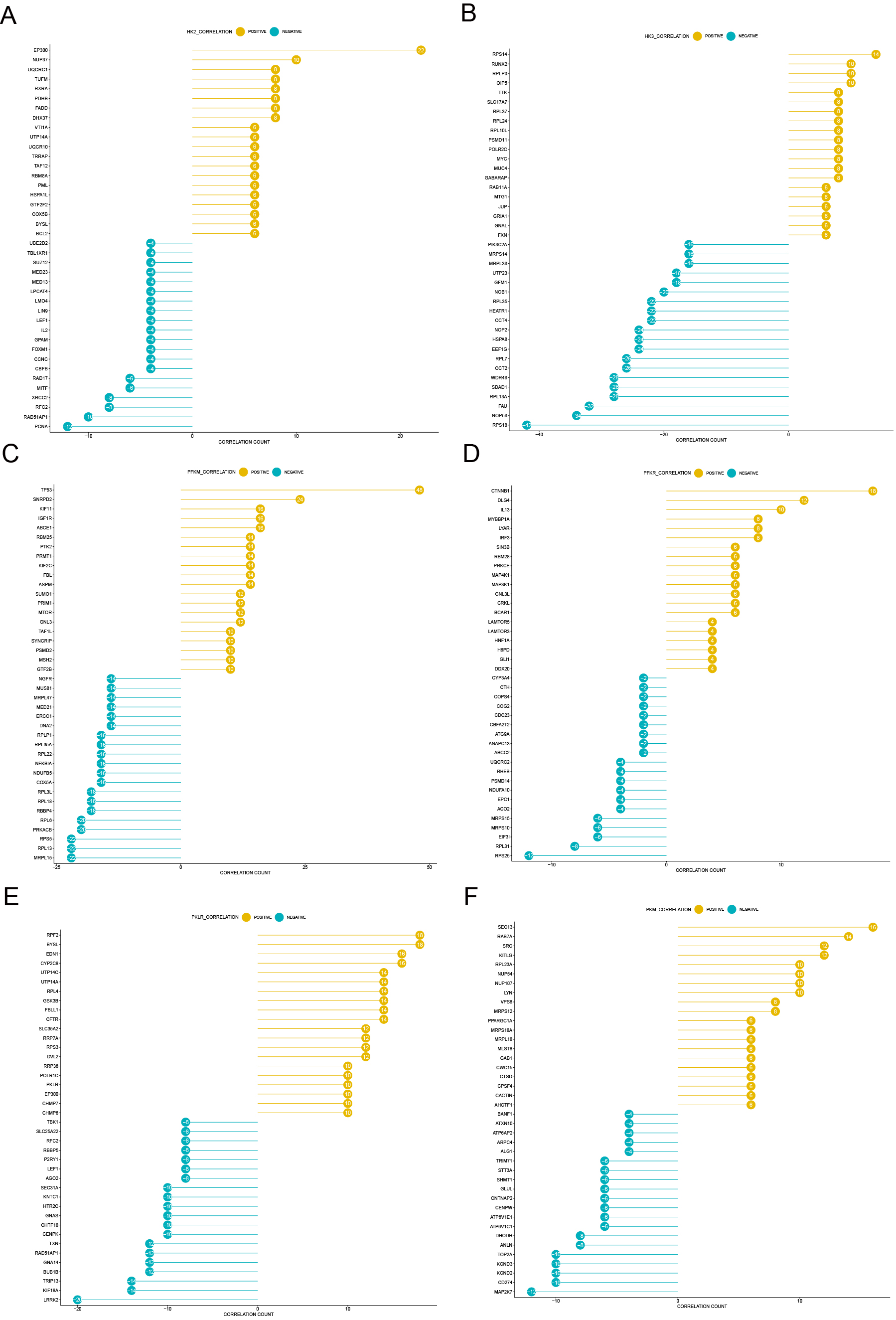

Supplement: Supplementary file 2 [file Image1.JPEG]

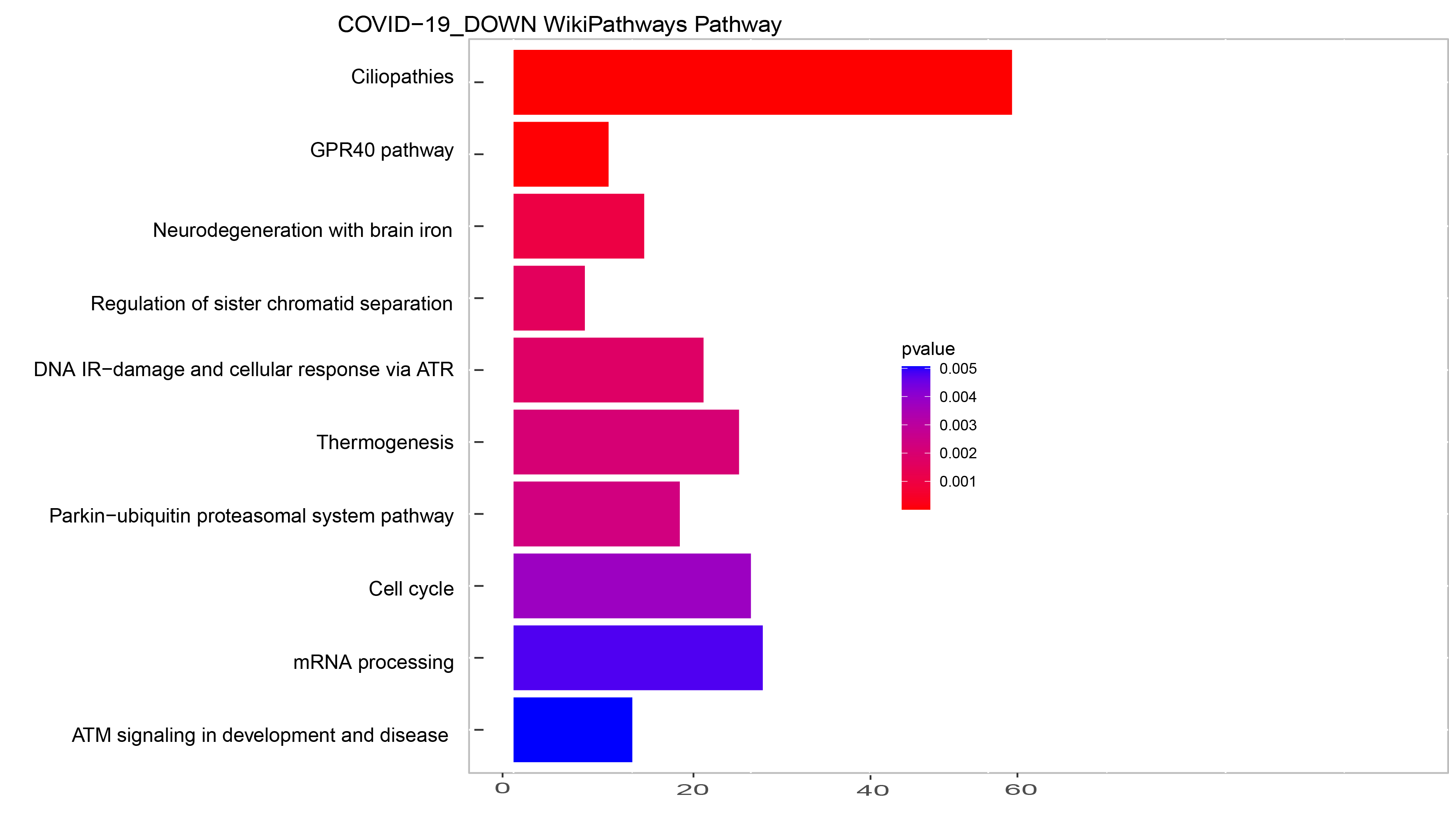

Supplement: Supplementary file 3 [file Image2.JPEG]
